# Effect of Thermoset Matrix Type on the Mechanical, Thermomechanical, and Electromagnetic Performance of Coremat Interlayered Carbon, Glass, and Aramid Fiber-Reinforced Hybrid Sandwich Composites

**DOI:** 10.3390/ma19194111

**Published:** 2026-09-25

**Authors:** Ersin Bahceci, Saim Kaltar

**Affiliations:** 1 Department of Metallurgy and Materials Engineering, Faculty of Engineering and Natural Sciences, Iskenderun Technical University, Hatay 31200, Türkiye; 2 Department of Metallurgy and Materials Engineering, Institute of Graduate Studies, Iskenderun Technical University, Hatay 31200, Türkiye; saimkaltar@gmail.com

**Keywords:** hybrid composites, carbon fiber, glass fiber, aramid fiber, coremat, electromagnetic shielding, electromagnetic absorption, mechanical properties

## Abstract

**Highlights:**

• Carbon-rich composites achieved high EMI shielding with very low transmission.
• Carbon/aramid hybrids enhanced true absorption while maintaining low transmission.
• Fiber matrix and hybrid architecture strongly affected multifunctional performance.
• Hybrid design enables tailoring of structural and electromagnetic performance.
• The composites are promising for defense, aerospace, automotive, and EV applications.
• The dataset provides a comparative reference for multifunctional composite design.

**Abstract:**

Fiber-reinforced polymer composites are attractive multifunctional material systems for aerospace, defense, and transportation because of their low density, high specific mechanical properties, and tailorable electromagnetic response. This study comparatively evaluated the effects of fiber system, thermoset matrix, and hybridization on the mechanical, thermomechanical, and electromagnetic behavior of Coremat interlayered sandwich composites containing carbon, glass, and aramid fabrics with polyester, vinylester, and epoxy matrices. Eighteen configurations were fabricated by hand lay-up and evaluated by tensile, three-point bending, heat deflection temperature (HDT), Vicat softening temperature (VST), and two-port S-parameter measurements over 4–40 GHz. The carbon/epoxy configuration exhibited the highest tensile strength (231.2 MPa), whereas the glass/vinylester configuration showed the highest flexural strength (160.5 MPa), HDT (128.0 °C), and VST (132.7 °C). The relatively low standard deviations across the tested configurations indicated consistent experimental repeatability and limited within-configuration dispersion. Carbon-containing systems strongly suppressed electromagnetic transmission; however, the single carbon systems were predominantly reflection driven. The carbon/glass polyester hybrid S10 provided the highest total shielding effectiveness (38.45 dB) at an overall laminate thickness of 3.30 mm, corresponding to a normalized shielding effectiveness of 11.65 dB/mm, while the carbon/aramid epoxy hybrid exhibited the highest incident-power absorption ratio (22.4%) together with very low transmission (0.22%). Overall, the results show that fiber system, matrix type, and hybrid architecture should be selected jointly when balancing structural, thermomechanical, and electromagnetic requirements.

## 1. Introduction

Fiber-reinforced polymer composites are widely used in aerospace, automotive, marine, and defense applications because of their low density, high specific mechanical properties, and design flexibility [1,2]. CFRPs combine high specific stiffness and strength with electrical conductivity [1,3], while glass fiber systems provide a favorable balance between cost and mechanical performance [4,5]. Aramid and glass/aramid hybrids can also offer advantages in specific strength and impact behavior [6], making hybrid laminate design an important approach for overcoming the limitations of single fiber systems [2,6,7].

The combination of structural and electromagnetic properties makes these composites attractive for multifunctional applications. Glass- and aramid-based composites are used in radome structures where electromagnetic transmission is critical [8], while glass and carbon fiber composites and sandwich/hybrid structures are being investigated for lightweight electric vehicle battery enclosures requiring mechanical, thermal, and electromagnetic functionality [9,10,11]. Their high specific strength and stiffness also support weight reduction and improved energy efficiency in transportation structures [10,12].

Composite mechanical performance depends not only on fiber properties but also on fiber volume fraction, stacking sequence, fiber–matrix interface, and manufacturing induced defects [2,4,5,7]. In hand lay-up processes, impregnation quality, voids, and resinrich regions can significantly affect laminate performance [5]. Therefore, the effects of epoxy, polyester, and vinylester matrices should be evaluated together with reinforcement architecture and manufacturing conditions.

Electromagnetic behavior is similarly governed by material and structural parameters. Carbon-based reinforcements can form conductive networks that strongly interact with electromagnetic waves, while their distribution, orientation, and structural organization within the matrix can significantly influence electromagnetic wave attenuation and shielding performance [1,13,14], while the response of CFRPs depends on conductivity, complex permittivity, fiber orientation, stacking sequence, thickness, frequency, and polarization [13,15,16,17]. Since incident electromagnetic energy is distributed among reflection, absorption, and transmission, total shielding effectiveness alone is insufficient to describe the shielding mechanism; S-parameters and relevant electromagnetic properties should therefore be considered together [13,15,18].

In sandwich composite structures, the core layer plays an important role in controlling laminate thickness, stiffness, and overall structural efficiency. Coremat, a nonwoven polyester-based core material, can increase the overall laminate thickness and stiffness without requiring the addition of multiple reinforcement layers, while its porous structure provides effective resin impregnation and integration with the surrounding fiber-reinforced skins [19]. Therefore, coremat was selected in the present study as an intermediate layer to establish a consistent sandwich architecture while limiting unnecessary reinforcement layer addition and to enable a systematic investigation of the combined effects of fiber type and thermoset matrix on the mechanical, thermomechanical, and electromagnetic responses of the resulting composites.

Previous studies have enhanced the electromagnetic performance of carbon fiber composites through CNT modification, conductive coatings, and other interface engineering approaches [20,21,22]. In contrast, electrically insulating glass fiber systems generally require conductive or magnetic secondary phases to achieve substantial EMI shielding [23,24,25]. Overall, the literature has predominantly focused on carbon/epoxy systems [13,15,16,17,18,20,21,22], whereas systematic comparisons involving carbon, glass, and aramid fibers with polyester, vinylester, and epoxy matrices remain limited.

Accordingly, a research gap remains in the simultaneous comparison of fiber sys-tem, thermoset matrix, and hybridization under a common manufacturing route and comparable coremat interlayered architecture. The present study addresses this gap by evaluating 18 single fiber and hybrid fiber sandwich configurations based on carbon, glass, and aramid fabrics with polyester, vinylester, and epoxy matrices. Mechanical, thermomechanical, and broadband electromagnetic responses over the 4–40 GHz frequency range, spanning the microwave and lower millimeter-wave regions, are assessed within the same experimental framework to identify configuration dependent tradeoffs. The purpose is not to assign the observed behavior to a single constituent in isolation, but to establish comparative structure property relationships for the complete laminate systems.

## 2. Materials and Methods

In the experimental study, 18 composite plate configurations were fabricated using carbon, glass, and aramid fabrics as reinforcement systems and polyester, vinylester, and epoxy resins as matrices. A coremat interlayer was incorporated in every configuration; therefore, coremat was treated as a controlled architectural element rather than an independent experimental variable. The laminates were designed as coremat interlayered sandwich structures, and the study focused on the combined effects of reinforcement system, matrix type, and hybridization. A 2 mm thick coremat Xi core interlayer was used as the sandwich core material in all composite configurations.

### 2.1. Materials

The materials used in composite fabrication were classified into three groups: reinforcement materials, resin systems, and core material. Material selection was based on mechanical performance and industrial applicability. Three different resins were used as matrix materials, vinylester (Polipol Polives™ 702 (Poliya, Istanbul, Türkiye)), polyester (Polipol™ 320-TA (Poliya, Istanbul, Türkiye)), and epoxy (HEXION LR635 (Hexion B.V., Rotterdam, The Netherlands)). Poliya COBALT (Poliya, Istanbul, Türkiye) (0.1–0.2%) was used as an accelerator, while MEKP (Poliya, Istanbul, Türkiye) (1–2%) was used as the curing agent for the polyester and vinylester resins. The HEXION LR635 (Hexion B.V., Rotterdam, The Netherlands) epoxy resin was supplied with the required curing component already incorporated by the manufacturer; therefore, no additional hardener was added and no separate resin to hardener mixing ratio was applied during fabrication. Polyester, vinylester, and epoxy resins were selected as three widely used but chemically and functionally distinct thermoset matrix systems to systematically evaluate the effect of matrix type on composite performance. Polyester (Polipol™ 320-TA(Poliya, Istanbul, Türkiye)) was used as a conventional and cost-effective matrix with favorable processability, while vinylester (Polipol Polives™ 702(Poliya, Istanbul, Türkiye)) was selected for its combination of good processability, mechanical performance, and interfacial characteristics. Epoxy (HEXION LR635) was included as a structural thermoset matrix generally associated with strong fiber matrix adhesion and high mechanical performance. The use of these three matrix systems enabled the matrix dependent behavior of carbon, glass, and aramid fiber composites and their hybrid architectures to be comparatively assessed under similar manufacturing and testing conditions.

The composites were reinforced with a 200 g/m^2^ twill woven carbon fiber fabric made from Tenax-E HTA 40 3K (Teijin Carbon Europe GmbH, Wuppertal, Germany) yarn, a 170 g/m^2^ twill woven aramid fabric made from KEVLAR^®^ 49 (DuPont, Wilmington, DE, USA) para aramid fibers, and a 390 g/m^2^ plain woven glass fiber fabric. According to the manufacturer data, the tensile strengths of the carbon, aramid, and glass fibers were 3950, 1990, and 3445 MPa, respectively, with corresponding elastic moduli of 238.1, and 73 GPa. These reinforcement materials were selected considering their low weight, mechanical performance, and resin compatibility. In addition, a low density core material was incorporated between the fiber-reinforced layers to form a sandwich structure, thereby increasing the laminate thickness and structural stiffness with a limited increase in overall weight. Because the reinforcing fabrics had different areal weights, the reported comparisons represent the performance of the complete laminate configurations rather than the intrinsic performance of the individual fiber types.

### 2.2. Method

The composite plates fabricated by hand lay-up were cut by waterjet according to the relevant standards. Mechanical properties were evaluated by tensile and three-point bending tests, while thermomechanical behavior was characterized by HDT and Vicat tests. The electromagnetic transmission characteristics of the composites were also investigated using a network analyzer.

#### 2.2.1. Composite Fabrication

Polyester, vinylester, and epoxy resins were used as matrix systems, while carbon, aramid, and glass fiber fabrics were employed as reinforcements. The compositions and stacking configurations of the specimens are summarized in Table 1. Composite sandwich plates with dimensions of 210 mm × 290 mm were fabricated by the hand lay-up method, with a 2 mm thick Coremat Xi interlayer(Lantor B.V., Veenendaal, The Netherlands) positioned between two reinforcement layers on each face. The reinforcement fabrics were arranged in a 0/90° orientation.

To minimize potential manufacturing induced defects associated with the hand lay-up process, including incomplete impregnation, resin rich or resin starved regions, air entrapment, and layer misalignment, all laminates were fabricated under strictly controlled and standardized processing conditions. All panels were manufactured in a climate controlled laboratory at 22 ± 2 °C and 45 ± 5% relative humidity using materials from the same respective batches. For each laminate configuration (S1–S18), the required matrix amount was determined by considering the areal weights of the reinforcement fabrics (200 g/m^2^ 3K plain weave carbon, 390 g/m^2^ 2/2 twill glass, and aramid fabrics) together with the resin absorption capacity of the 2 mm Coremat Xi interlayer (1.2 kg/m^2^). The required resin quantities were weighed using a digital balance with an accuracy of ±0.001 g.

During lay-up, the reinforcement fabrics were thoroughly impregnated with the corresponding resin system, and uniform manual pressure was applied from the center toward the edges using serrated aluminum consolidation rollers. This procedure facilitated the removal of entrapped air between adjacent layers and promoted uniform resin penetration throughout the fiber architecture. Alignment guides positioned along the mold boundaries were used to minimize deviations in fiber orientation and maintain the prescribed laminate architecture.

Following fabrication, the laminates were subjected to matrix specific curing and post curing procedures to promote complete crosslinking and reduce residual internal stresses. Polyester matrix laminates were initially cured at room temperature for 24 h and subsequently post cured at 80 °C for 2 h. Vinylester matrix laminates were subjected to a stepwise post curing treatment at 80 °C for 3 h followed by 100 °C for 1 h, whereas epoxy matrix laminates were post cured in an oven at 60 °C for 15 h. The resulting laminates exhibited relatively low void contents, ranging from 2.1% to 4.2%, indicating effective impregnation and consolidation under the standardized fabrication conditions. These controlled manufacturing procedures also contributed to the repeatability of the subsequent mechanical and thermomechanical measurements.

After curing, test specimens were prepared from the fabricated plates according to the dimensions required for the respective characterization methods: 20 × 150 mm for tensile testing, 100 × 10 mm for three-point bending, 120 × 10 mm for heat deflection temperature (HDT) testing, and 25 × 25 mm for Vicat softening temperature (VST) testing.

#### 2.2.2. Tensile Test

Tensile tests were performed according to TS EN ISO 527-4 [26] at a crosshead speed of 2 mm/min. No end tabs were used; the specimens were directly clamped in the testing machine grips. Five specimens from each configuration were tested. The tensile results are reported as mean ± standard deviation (SD, *n* = 5) to account for specimen level experimental variability, and the corresponding statistical analyses were performed to evaluate differences among the configurations.

#### 2.2.3. Three Point Bending Test

Three-point flexural tests were conducted using an AUTOGRAPH AG-IS 20 kN (Shimadzu Corporation, Kyoto, Japan) universal testing machine in accordance with EN ISO 14125 [27], following Method A (three-point loading) for Class II materials (woven fabric reinforced composites). For specimens with a nominal thickness (h) of 3 mm, the support span (L) was set to 48 mm and the test speed to 2 mm/min. The radius of the central loading member (R_1_) was 5 ± 0.2 mm, while the radius of the supports (R_2_) was 2 ± 0.2 mm. Five specimens were tested for each composite configuration.

#### 2.2.4. Heat Deflection Temperature (HDT) Test

HDT tests were performed according to ISO 75 [28] using a CEAST HDT-VICAT (Instron, Norwood, MA, USA) system and specimens measuring 120 × 10 × 3(+0.5) mm. A constant stress of 1.80 MPa was applied in a silicone oil bath while the temperature was increased at 120 °C/h. The temperature corresponding to the specified deflection was recorded as the HDT value.

#### 2.2.5. Vicat Softening Temperature (VST) Test

Vicat tests were performed according to TS EN ISO 306 [29]. The specimens were conditioned in an oil bath at an initial temperature of 27 °C for 5 min, followed by testing according to the Method B50 procedure.

#### 2.2.6. Electromagnetic Interference (EMI) Shielding Characterization Using S-Parameters

Electromagnetic measurements were performed over the 4–40 GHz frequency range using an R&S^®^ ZNB40 Vector Network Analyzer (VNA; 10 MHz–40 GHz, two test ports, 2.92 mm connectors, Order No. 1311.6010K72; Rohde & Schwarz GmbH & Co. KG, Munich, Germany) with a free space horn antenna configuration (Figure 1). Clear site reference measurements were used for calibration, and S_11_, S_21_, S_12_, and S_22_ parameters were recorded at 3601 frequency points. The measurements were conducted under controlled laboratory conditions at a temperature of 23 ± 2 °C and a relative humidity of 50 ± 5% RH.

Electromagnetic measurements were performed over the 4–40 GHz frequency range using a CTG free space focused beam measurement system equipped with a pair of MVG QH4000 open boundary Rohde & Schwarz GmbH & Co. KG, Munich, Germany) quad ridge horn antennas and dielectric focusing lenses. The dielectric lenses focused the electromagnetic field onto the specimen region, improving spatial confinement and reducing edge related diffraction effects. To further isolate the specimen response, time domain gating was applied to the measured S-parameters to suppress temporally separated unwanted reflections and multipath contributions. A 1 ns symmetric time gate was used for all measurements, with the metal reference plate employed during calibration defining the time zero/reference position for the gating procedure. The selected gate width was within the 0.5–1.0 ns range recommended by the CTG manufacturer for typical free-space measurements.

Electromagnetic measurements were performed using a focused-beam configuration with dielectric focusing lenses to concentrate the incident field primarily on the central region of the specimen and reduce edge-related contributions. No additional RF absorbing baffles were used. A 1 ns symmetric time domain gate was independently applied to suppress delayed reflections and multipath contributions; however, finite-size effects may be more pronounced near the lower-frequency limit because of the wavelength to specimen size ratio.

The electromagnetic behavior of the 18 composite specimens was reevaluated based on the S_11_, S_21_, S_12_, and S_22_ scattering parameters (S-parameters) measured over the 4–40 GHz frequency range. S_11_ and S_22_ represent reflection for excitation from Port 1 and Port 2, respectively, whereas S_21_ and S_12_ represent forward and reverse transmission, respectively. The amplitude ratios obtained from the vector network analyzer in decibels (dB) were converted into power coefficients, and the reflection (R), transmission (T), true absorption (A), and total shielding effectiveness (SE_T_) were calculated. This approach is consistent with the literature concerning the use of S-parameters to distinguish the mechanisms contributing to electromagnetic interference (EMI) shielding [30,31,32,33,34,35,36]. Mikinka and Siwak [13] demonstrated that the shielding performance of CFRP composites is strongly dependent on structural variables such as carbon fiber content, fiber geometry, and, particularly in continuous fiber-reinforced structures, the stacking sequence. Isari et al. [37] further emphasized that impedance matching, multiple internal reflections, dielectric/magnetic losses, and structural design should be considered collectively when designing absorption dominant EMI shielding materials.

(1) ***S*****_ij,dB_ = 20 log_10_ |S_ij_|**,

where **S_ij,dB_** represents the amplitude of the scattering parameter expressed in decibels, and |**S_ij_**| denotes the absolute magnitude ratio of the corresponding complex scattering parameter.

(2) ***R*****_1_ = |S_11_|^2^ = 10^(S11,dB/10)^**,

where **R_1_** is the dimensionless fraction of the total electromagnetic power incident from Port 1 that is reflected back from the specimen.

(3) *T*_21_ = |S_21_|^2^ = 10^(S21,dB/10)^,

where **T_21_** is the ratio of the electromagnetic power transmitted from Port 1 to Port 2 to the total incident electromagnetic power.

(4) ***A*****_1_ = 1 − R_1_ − T_21_**,

where **A_1_** represents the true absorption ratio, i.e., the fraction of the total incident electromagnetic power dissipated within the specimen. For a passive system, the energy balance condition **R_1_ + T_21_ + A_1_ =** **1** must be satisfied.

(5) ***SE*****_T,21_ = −10 log_10_(T_21_) = −S_21,dB_**,

where **SE_T,21_** is the total electromagnetic shielding effectiveness in the Port 1 to Port 2 direction and is expressed in decibels (dB).

(6) ***SE*****_R,1_ = −10 log_10_(1 − R_1_)**,

where **SE_R,1_** is the effective shielding component attributed to reflection at the first surface.

(7) ***SE*****_A,1_ = −10 log_10_[T_21_/(1 − R_1_)]**,

where **SE_A,1_** is the effective absorption shielding component defined with respect to the electromagnetic energy that is not reflected at the first surface and therefore enters the specimen.

(8) ***A*****_eff,1_ = A_1_/(1 − R_1_)**,

where **A_eff,1_** represents the fraction of the electromagnetic energy that is not reflected at the first surface and enters the specimen but is subsequently dissipated within the material.

For excitation from the Port 2 direction, the same calculation procedure was applied using ***R_2_*** ***= |S_22_|^2^, T_12_ = |S_12_|^2^***, and ***A_2_ = 1 − R_2_ − T_12_***. Particularly for asymmetric hybrid stacking sequences, S_11_ and S_22_ should not necessarily be expected to be equal. Hwang et al. [31] emphasized that a high total shielding effectiveness alone does not demonstrate absorption dominant behavior and that the actual shielding mechanism should be determined by evaluating ***R, T,*** and ***A*** collectively. Similarly, Isari et al. [37] stated that identifying the shielding mechanism solely on the basis of total shielding effectiveness may be misleading and that reflection and absorption should be evaluated separately.

## 3. Results and Discussion

### 3.1. Physical and Compositional Characteristics of the Composite Configurations

The physical and compositional characteristics presented in Table 2 demonstrate the effects of matrix type and reinforcement architecture on the resulting composite structures. The relatively narrow range of average thicknesses across all configurations (3.08–3.45 mm), together with standard deviations of only 0.05–0.10 mm, indicates acceptable geometrical consistency among the hand lay-up fabricated laminates. The lowest average thickness was obtained for S3 (3.08 ± 0.05 mm), whereas S4 exhibited the highest value (3.45 ± 0.10 mm). Within a given reinforcement architecture, a general decrease in both laminate thickness and estimated void fraction was observed from polyester to vinylester and epoxy matrices, suggesting that matrix type influenced fiber wetting, compaction, and laminate consolidation during fabrication.

The experimental density ranged from 1.15 to 1.42 g/cm^3^ and was strongly dependent on the reinforcement type. Glass/glass configurations exhibited the highest densities (1.38–1.42 g/cm^3^), whereas aramid/aramid and carbon/aramid systems showed comparatively lower values. The fiber mass fraction varied between 28.5 and 43.1%, while the fiber volume fraction ranged from 21.2 to 26.1%. The higher fiber mass fractions observed for the glass/glass specimens are consistent with the differences in density among the reinforcing fibers; therefore, direct performance comparisons based solely on mass fraction should be interpreted with caution.

The estimated void fractions remained within 2.5–3.8% for all specimens, with epoxy-based configurations generally exhibiting the lowest values within each reinforcement architecture. For example, the estimated void fraction decreased from 3.2% for the polyester-based carbon/carbon specimen S1 to 2.5% for the corresponding epoxy-based S3, while a decrease from 3.8% for S4 to 2.8% for S6 was observed in the glass/glass group. Similar trends were evident in the hybrid configurations. These results indicate that, in addition to matrix chemistry, laminate consolidation and void content should be considered when interpreting the mechanical and thermomechanical responses. Nevertheless, the relatively limited variation in fiber volume fraction and the comparable laminate thicknesses across the configurations support the interpretation of matrix type and reinforcement/hybrid architecture as the principal design variables when discussing the subsequent mechanical, thermomechanical, and electromagnetic performance.

### 3.2. Tensile Test

The tensile results are presented in Figure 2. The highest reported tensile strength was obtained for S3 (231.2 MPa), whereas S7 exhibited the lowest value (106.4 MPa). The elastic modulus ranged from 2818 to 10243 MPa. On a descriptive basis, the carbon/carbon group showed the highest average tensile strength (194.4 MPa), compared with the glass/glass (169.3 MPa) and aramid/aramid (145.5 MPa) groups. Because the fabric areal masses and fiber volume fractions differ among reinforcement systems, these values describe the response of the complete laminate configurations and should not be interpreted as an isolated fiber property ranking [38,39]. As shown in Table 2, S3 combined a relatively high fiber volume fraction (22.5%) with the lowest estimated void fraction (2.5%), which may have contributed to improved laminate consolidation and tensile load transfer. Consistent with the overall stress–strain behavior presented in Figure 2, Figure 3a provides a clearer comparison of the characteristic tensile responses among the configuration groups using representative curves. These curves correspond to the specimens exhibiting the median tensile strength within each three-specimen group (S2, S4, S8, S10, S13, and S18), thereby minimizing the influence of extreme responses on the comparative assessment. This selection reduces the visual influence of extreme tensile responses and provides a representative comparison of the characteristic stress–strain behavior of each configuration group, while the quantitative and statistical evaluations were performed using the complete dataset of all 18 configurations through the Mean ± SD values and ANOVA/Tukey HSD analyses. The statistical representation in Figure 3b (and Table 3) shows that S3 exhibited the highest mean tensile strength (231.2 ± 16.6 MPa), whereas S7 showed the lowest mean value (106.3 ± 7.1 MPa). Across all 18 configurations, the SD values ranged from 7.1 to 16.6 MPa, indicating a relatively consistent level of within-configuration experimental dispersion. Tukey HSD identified significant differences in 87 of the 153 pairwise comparisons (*p* < 0.05). However, the ANOVA result in Table 4 represents differences among the complete laminate configurations rather than an isolated main effect of matrix type.

The tensile response also varied systematically with matrix type when the configurations were compared descriptively. Considering all specimens, the average maximum tensile stresses were approximately 182.6, 153.8, and 147.0 MPa for epoxy, vinylester, and polyester matrices, respectively. In particular, the carbon/Coremat/carbon specimen S3 reached 231.2 MPa with the epoxy matrix, indicating more effective load transfer in the carbon reinforced structure [40]. The generally lower void fractions of the epoxy-based specimens further support the contribution of improved consolidation to their tensile response. The error-bar representation in Figure 3b further demonstrates that the comparatively high tensile response of S3 was retained after accounting for experimental dispersion (231.2 ± 16.6 MPa). Nevertheless, the configuration-level ANOVA should not be interpreted as demonstrating an independent statistically significant main effect of matrix type; rather, the matrix-related trend should be considered within the combined laminate architecture. Accordingly, the ANOVA and Tukey HSD results are used here to assess differences among the 18 complete laminate configurations (Table 3 and Table 4).

Among the hybrid systems, the average maximum tensile stresses of the carbon/glass, carbon/aramid, and glass/aramid groups were approximately 157.0, 157.4, and 143.3 MPa, respectively. The carbon/aramid epoxy specimen S15 was particularly notable, combining a high tensile strength of 212.9 MPa with a strain of 0.103 mm/mm. This behavior can be attributed to the combination of the high strength of carbon fibers and the higher strain and energy absorption capability of aramid fibers [41,42]. S15 also exhibited a low estimated void fraction (2.6%), consistent with adequate laminate consolidation. The representative curves in Figure 3a complement the complete set of curves in Figure 2 by showing that the differences among the configuration groups were not restricted to maximum tensile strength but were also reflected in their deformation to failure behavior. Differences in curve extension and in the progression toward final failure indicate a configuration-dependent tensile response. Consistent with its individual stress–strain response, S15 maintained a high mean tensile strength of 212.9 ± 15.8 MPa in Figure 3b. The Tukey HSD groups in Table 3 provide the statistical basis for comparisons among the individual configurations.

Overall, the results demonstrate that tensile behavior is governed not only by matrix and fiber type but also by hybridization, stacking sequence, and fiber–matrix interfacial characteristics [42,43,44,45,46]. While Figure 2 provides the complete stress–strain responses of all 18 specimens, Figure 3a facilitates comparison of the characteristic tensile behavior through representative curves from each configuration group, and Figure 3b provides the corresponding statistical dispersion of maximum tensile strength through mean ± SD values. The ANOVA results (Table 4) and Tukey HSD comparisons (Table 3) statistically support this configuration-dependent tensile behavior and complement the mechanical interpretation of the combined effects of matrix, reinforcement type, and hybrid architecture. Taken together, the complete stress–strain curves, representative group responses, error-bar analysis, and configuration-level statistical comparisons indicate that the observed differences in tensile performance reflect systematic differences among the complete laminate configurations rather than being attributable solely to experimental scatter.

### 3.3. Fracture Analysis

Figure 4, Figure 5 and Figure 6 show the macroscopic fracture and damage morphologies of the specimens after tensile testing. Evaluation of the tensile results together with the fracture images indicates that the mechanical behavior was governed by resin type, fiber type, fiber–matrix interfacial behavior, and hybridization. In general, the epoxy matrix provided higher strength and more controlled damage development, particularly in carbon-containing systems. Carbon fibers promoted localized fracture because of their high stiffness and load bearing capacity, whereas aramid fibers contributed to progressive damage and energy absorption through fibrillation and fiber pull out. Glass fibers exhibited a comparatively more distributed deformation and damage behavior. The physical characteristics in Table 2 further show that the epoxy-based specimens generally had lower estimated void fractions, which is consistent with their more localized damage and better preservation of interlaminar integrity.

The fracture morphologies of S1–S6, comprising the carbon/carbon and glass/glass systems, are presented in Figure 4. Carbon/polyester S1 exhibited pronounced delamination, matrix damage, and localized carbon bundle fracture, whereas damage in vinylester-based S2 was mainly characterized by Coremat crushing and limited interfacial separation. In epoxy-based S3, the face Coremat interface remained largely intact and damage was concentrated within the core and localized carbon fiber fracture zones. This more integrated damage mechanism is consistent with the highest tensile strength of 231.2 MPa obtained for S3 (Figure 2). Notably, S3 also exhibited the lowest estimated void fraction (2.5%), consistent with its preserved interfacial integrity. Among the glass reinforced specimens S4–S6, polyester-based S4 showed more extensive delamination, matrix cracking, and fiber pull out, while damage was more localized in vinylester-based S5. In epoxy-based S6, layer integrity was largely maintained and damage occurred predominantly within the Coremat.

The fracture behavior of the aramid/aramid and carbon/glass specimens S7–S12 is shown in Figure 6. Polyester–aramid S7 exhibited extensive interfacial separation, aramid fibrillation, and fiber pull out, whereas vinylester-based S8 retained greater layer integrity despite the persistence of fibrillation and core damage. Epoxy–aramid S9 was characterized by localized fiber deformation and cohesive Coremat damage rather than extensive delamination. The carbon/glass hybrids S10–S12 exhibited asymmetric damage due to the different deformation characteristics of carbon and glass fibers. Interfacial and core damage was more pronounced in polyester-based S10, while vinylester S11 showed improved layer integrity. Epoxy-based S12 exhibited the greatest preservation of interlaminar integrity, with damage mainly localized within the matrix/core region. This progression is also consistent with the decrease in estimated void fraction from S10 (3.5%) to S12 (2.6%).

The carbon/aramid and glass/aramid hybrid morphologies of S13–S18 are presented in Figure 5. In the carbon/aramid group, S13 and S14 largely maintained the overall sandwich integrity, whereas epoxy-based S15 exhibited improved face–Coremat integrity and localized aramid fibrillation. The combination of a tensile strength of 212.9 MPa and a strain of 0.103 mm/mm for S15 (Figure 2) demonstrates the effective combination of the high load bearing capability of carbon fibers with the deformation and energy absorption capacity of aramid fibers. The relatively low estimated void fraction of S15 (2.6%) is also consistent with its improved face coremat integrity. In the glass/aramid group, S16 and S17 exhibited more distributed and asymmetric damage, whereas epoxy-based S18 showed better preservation of interlaminar integrity, with damage predominantly localized within the coremat.

Overall, the fracture morphologies support the mechanical test results. The carbon/epoxy system was the most advantageous in terms of maximum tensile strength, whereas the carbon/aramid–epoxy hybrid provided the most favorable balance between strength, deformation capacity, and damage tolerance. The ability of the epoxy matrix to limit extensive interfacial separation and promote localized or cohesive core damage in several configurations suggests more effective load transfer. Together with the lower estimated void fractions of the epoxy-based configurations (Table 2), these observations indicate that laminate consolidation may also have contributed to the observed fracture behavior. However, the favorable performance of vinylester in some glass-containing systems also confirms that optimum composite performance depends not only on the individual resin or fiber properties but also on fiber matrix compatibility, hybrid stacking configuration, and load sharing mechanisms between the constituent layers.

The sawtooth like response observed in the stress/strain curves of S2, S3, and S15 was reproducible across multiple specimens and experimental sets rather than being limited to a single measurement. No visible specimen slippage at the grips was observed during these tests. The repeated occurrence of successive stress drops and recoveries therefore suggests a progressive damage response of the composite structure, potentially involving matrix cracking, local fiber matrix interfacial debonding, progressive fiber/bundle failure, and subsequent load redistribution among the remaining intact fibers. Accordingly, the sawtooth characteristics were interpreted as part of the progressive tensile damage behavior rather than as an isolated experimental artifact.

### 3.4. Three-Point Bending Test Results

The three-point bending results showed that flexural behavior was strongly influenced by resin type, fiber type, and hybrid configuration. The maximum flexural stress values, expressed as mean ± standard deviation (SD), together with the Tukey HSD post hoc comparisons, are presented in Table 5. The highest flexural strength among all specimens was obtained for the vinylester-based glass/Coremat/glass specimen S5 at 160.4 ± 8.0 MPa, followed by carbon/Coremat/carbon vinylester S2 (147.1 ± 4.2 MPa) and carbon/Coremat/carbon epoxy S3 (136.6 ± 6.8 MPa) (Table 5). The lowest value was recorded for aramid/Coremat/aramid vinylester S8 at 55.5 ± 2.7 MPa. Tukey HSD analysis (α = 0.05) confirmed statistically significant differences among several configurations (Table 5), demonstrating that the observed variations in flexural performance were not solely attributable to within-group experimental variation. These results indicate that flexural performance depends not only on fiber tensile strength but also on fiber matrix compatibility, the behavior of the tensile and compressive faces, and interlaminar shear resistance. As shown in Table 2, S5 also exhibited a relatively high fiber volume fraction (25.6%) and a low estimated void fraction (3.0%), which are consistent with effective reinforcement utilization and laminate consolidation under flexural loading.

The effect of resin type was distinct but dependent on the fiber configuration. Vinylester provided the highest flexural strength in the carbon/carbon and glass/glass groups, whereas epoxy was superior in the aramid/aramid, carbon/glass, and glass/aramid systems. In the carbon/aramid group, the highest value was obtained with the polyester matrix. Therefore, no single resin system was superior for all configurations, confirming the importance of resin–fiber compatibility in flexural performance. This observation was statistically supported by the two-way ANOVA results (Table 6), matrix resin type had a significant effect on maximum flexural stress [F(2,72) = 8.65, *p* < 0.001], while the matrix × reinforcement architecture interaction was also highly significant [F(10,72) = 38.33, *p* < 0.001]. The corresponding partial eta-squared (ηp^2^) values were 0.194 and 0.842, respectively, confirming that the influence of the matrix strongly depended on the reinforcement architecture. Notably, S6 had a slightly higher fiber volume fraction (26.1%) and lower estimated void fraction (2.8%) than S5, yet did not achieve the same flexural strength, indicating that these physical parameters alone cannot explain the bending response.

Regarding fiber type, the most notable result was the superior performance of the glass reinforced S5 specimen. Although the carbon/carbon–epoxy S3 specimen exhibited the best tensile performance, its flexural strength was limited to 136.6 MPa. This difference can be attributed to the combined tensile, compressive, and shear stresses generated during bending. Thus, flexural behavior is governed not only by fiber tensile strength but also by compressive response and interlaminar bonding. This interpretation is further supported by the ANOVA results, which identified reinforcement architecture as the dominant statistical factor affecting flexural performance [F(5,72) = 315.74, *p* < 0.001, ηp^2^ = 0.956] (Table 6). Overall, the statistical analysis confirms that flexural performance is governed primarily by the specific matrix–reinforcement combination rather than by either constituent independently.

The main contribution of aramid-containing systems was observed in deformation capacity rather than maximum flexural strength. Although S7 exhibited a flexural strength of only 81.8 MPa, it reached the highest flexural strain among all specimens (5.62%) and a maximum displacement of 7.8 mm. Similarly, carbon/aramid–epoxy S15 exhibited 116.3 MPa flexural strength together with 4.2 mm displacement and 2.76% strain, while glass/aramid–polyester S16 reached 5.1 mm displacement and 3.25% strain despite its lower flexural strength of 78.8 MPa. These results suggest that aramid-containing hybrid systems improve deformation capacity and damage tolerance by restricting crack propagation and maintaining load transfer after initial damage.

Post test observations were consistent with these numerical results. Rather than sudden and complete fracture, the specimens predominantly exhibited permanent deformation beneath the loading axis, accompanied by matrix cracking and localized delamination. The overall integrity of the carbon and glass reinforced specimens was largely preserved, indicating that the composites dissipated flexural energy through progressive damage mechanisms rather than purely brittle fracture. Overall, the vinylester glass fiber system provided the highest flexural strength, whereas aramid-containing hybrid systems offered superior deformation capacity and damage tolerance.

### 3.5. Heat Deflection Temperature (HDT) Test Results

The effect of hybridization on HDT is summarized in Table 7. Carbon/aramid hybrids exhibited higher HDT values than carbon/glass hybrids for all resin systems. For polyester, vinylester, and epoxy matrices, the carbon/aramid hybrids reached 76.2, 80.7, and 72.3 °C, respectively, compared with 71.7, 73.0, and 57.8 °C for the corresponding carbon/glass hybrids. The difference was particularly pronounced in the epoxy system, where carbon/aramid hybridization increased HDT by approximately 25% relative to the carbon/glass configuration. Glass/aramid hybrids exhibited intermediate values. As shown in Table 2, the hybrid specimens exhibited relatively comparable fiber volume fractions (22.8–25.8%) and low estimated void fractions (2.6–3.7%); therefore, the observed HDT differences appear to be more closely associated with matrix–reinforcement interactions and hybrid architecture than with variations in fiber content alone.

However, the highest HDT among all specimens was obtained for the non-hybrid glass/glass vinylester system at 128.0 °C (Table 7). This indicates that HDT is governed not by hybridization alone, but more strongly by fiber matrix compatibility. Thus, carbon/aramid showed the best thermomechanical performance among the hybrid systems, whereas the glass fiber vinylester combination provided the highest overall dimensional stability at elevated temperature. This trend is also consistent with the HDT range reported for hybrid vinylester composites by Vignesh et al. [47].

### 3.6. Vicat Softening Temperature (VST) Test

The Vicat softening temperature (VST) values obtained according to TS EN ISO 306 Method B50 [29] ranged from 58.8 to 132.7 °C (Table 8). The highest VST was obtained for the glass/glass vinylester specimen S5 (132.7 °C), whereas the lowest was recorded for the glass/glass epoxy specimen S6 (58.8 °C). The 73.9 °C difference resulting solely from changing the matrix within the same reinforcement architecture demonstrates the strong influence of resin fiber compatibility. Notably, S5 and S6 had comparable fiber volume fractions (25.6 and 26.1%) and estimated void fractions (3.0 and 2.8%), respectively (Table 2), indicating that their large VST difference cannot be explained by fiber content or void fraction alone and further emphasizing the role of matrix reinforcement interactions. Similarly, in the carbon/carbon group, VST values of 82.1, 120.4, and 71.5 °C were obtained for polyester, vinylester, and epoxy matrices, respectively, with vinylester providing the greatest resistance to softening.

However, VST was also strongly influenced by hybridization. In the carbon/aramid group, S13, S14, and S15 exhibited VST values of 108.4, 125.9, and 131.5 °C, respectively (Table 8). Particularly, the VST of epoxy-based S15 (131.5 °C) was 72.7 °C higher than that of the glass/glass epoxy S6 specimen (58.8 °C), corresponding to an absolute temperature difference of ΔT = 72.7 °C. This pronounced difference indicates that the carbon/aramid hybrid architecture substantially improved the resistance of the epoxy system to softening at elevated temperatures, emphasizing the importance of fiber matrix interactions and hybrid architecture in thermomechanical behavior. The relatively low estimated void fraction of S15 (2.6%) is also consistent with good laminate consolidation, although the VST trend indicates that hybrid architecture and matrix reinforcement compatibility remain the dominant factors.

Macroscopic examination after testing (Figure 7) showed that the specimens largely maintained their geometry and physical integrity without visible thermal degradation, charring, or pronounced discoloration. Deformation remained localized around the indenter contact region, indicating that the measured VST primarily represents temperature dependent matrix softening and the resistance provided by the reinforcement structure rather than general thermal degradation. Overall, glass/glass vinylester S5 (132.7 °C) and carbon/aramid epoxy S15 (131.5 °C) emerged as the two most resistant configurations to softening at elevated temperatures.

### 3.7. Electromagnetic S-Parameters, Reflection, Transmission, and Absorption Behavior

The band averaged **R_1_, T_21_, A_1_, SE_T_**, and **A_eff_** values, calculated using Equations (2)–(8), are collectively presented in Table 9. Table 9 clearly demonstrates that total shielding effectiveness and the true absorption ratio do not represent the same physical quantity, and that the performance ranking of the specimens varies depending on the evaluation metric selected.

The electromagnetic behavior of the 18 composites was evaluated from the S_11_, S_21_, S_12_, and S_22_ parameters measured using a two-port VNA over 4–40 GHz. For the carbon fiber specimens S1–S3, transmission was very low and reflection was dominant (Figure 8, Figure 9 and Figure 10). As shown in Table 9, S1, S2, and S3 exhibited average reflection ratios of 95.11%, 94.37%, and 94.25%, respectively, while transmission remained at only 0.268%, 0.170%, and 0.254%. This strong shielding behavior is consistent with the EMI shielding potential of carbon fiber composites reported by Mikinka and Siwak [13]. However, their low true absorption ratios (4.62–5.49%) demonstrate that shielding was predominantly reflection driven rather than absorption driven, consistent with the interpretation proposed by Hwang et al. [31].

The glass fiber-reinforced specimens S4–S6 exhibited markedly higher electromagnetic transmission than the carbon fiber-reinforced group (Figure 11, Figure 12 and Figure 13). The power transmission coefficient (T_21_) ranged from 58.88% to 63.57%, while the true absorption ratio (A_1_) increased from 5.25% to 9.17% as the matrix changed from polyester to epoxy. Within this group, the higher true absorption exhibited by the epoxy matrix S6 specimen suggests that the matrix and fiber–matrix interface may play an important role in dielectric loss mechanisms [1,3].

For the aramid fiber-reinforced specimens S7–S9 shown in Figure 14, Figure 15 and Figure 16, the power reflection coefficient (R_1_) was relatively low, ranging from 13.90% to 18.52%, whereas the power transmission coefficient (T_21_) was relatively high, ranging from 69.03% to 73.39%. Nevertheless, the true absorption ratio (A_1_) ranged from 11.63% to 12.90%, corresponding to values more than approximately twice those observed for the single fiber carbon systems. This behavior indicates that a reduction in surface reflection facilitates the penetration of electromagnetic waves into the material, thereby promoting electromagnetic energy dissipation through mechanisms such as dielectric losses and interfacial polarization [1,3].

The matrix effect was particularly pronounced in the carbon/glass hybrids (Figure 17, Figure 18 and Figure 19). S10 and S12 exhibited high reflection (R_1_ = 91.63% and 93.85%, respectively) and comparatively low true absorption (A_1_ = 7.44% and 5.82%), whereas S11 showed lower reflection (79.45%) and substantially higher true absorption (18.93%). Thus, within the carbon/glass hybrid architecture, the vinylester matrix shifted the electromagnetic response toward greater incident power absorption, while the polyester-based S10 combined very low transmission (T_21_ = 0.93%) with the highest total shielding effectiveness among all investigated configurations (SE_T_ = 38.45 dB). This distinction further demonstrates that total shielding effectiveness and true absorption describe different aspects of electromagnetic performance: S10 was the most effective in suppressing transmission, whereas S11 dissipated a larger fraction of the incident electromagnetic energy within the material.

As shown in Table 9 and Figure 20, Figure 21 and Figure 22, the carbon/aramid hybrids combined low electromagnetic transmission with higher true absorption than the single carbon fiber composites. The A_1_ values of S13, S14, and S15 were 19.18%, 19.11%, and 22.40%, respectively, with S15 exhibiting the highest value among all 18 specimens. For S15, a reflection ratio of 77.39% and a very low transmission ratio of 0.22% indicate that hybridization redistributed the incident electromagnetic energy toward greater absorption compared with S1–S3. Such behavior is consistent with the influence of composite architecture on electromagnetic shielding and loss processes reported in the literature [1,8]. However, since complex permittivity, permeability, and impedance matching were not measured, this improvement should be attributed to the experimentally observed effect of hybrid architecture rather than to a specific loss mechanism.

Despite its highest A_1_ value (22.40%), S15 cannot be classified as absorption dominant because its reflection remained 77.39%. According to the A > 0.5 criterion proposed by Hwang et al. [31], its main advantage is therefore the combination of reduced reflection, increased true absorption, and very low electromagnetic transmission rather than absorption dominant shielding.

As shown in Figure 23, Figure 24 and Figure 25, the glass/aramid hybrids exhibited higher electromagnetic transmission than the carbon-containing hybrids, while true absorption increased from S16 (8.20%) to S17 (10.47%) and S18 (15.38%). Epoxy-based S18 showed the highest true absorption among the carbon free specimens, indicating that appropriate matrix selection and hybrid interfacial architecture can enhance electromagnetic energy dissipation even without conductive carbon fibers.

As shown in Figure 26, changing the matrix significantly affected the S_21_ response of the carbon/glass hybrids. Despite its higher transmission, S11 exhibited lower reflection and consequently higher true absorption than S10 and S12.

In the carbon/aramid group, all specimens exhibited very low S_21_ levels (Figure 27). S15 showed the lowest average T_21_ and the highest A_1_, indicating that the epoxy matrix effectively reduced electromagnetic transmission while enhancing energy dissipation.

In the glass/aramid group (Figure 28), S18 exhibited the lowest S_21_ response, with an average T_21_ of 59.44% and an A_1_ of 15.38%, demonstrating the superior electromagnetic performance of the epoxy matrix within this group.

In Figure 29, the polyester matrix hybrids S10, S13, and S16 are compared. Carbon/aramid S13 exhibited lower reflection and higher true absorption than carbon/glass S10, whereas glass/aramid S16 showed substantially higher transmission. Thus, the hybrid fiber combination strongly influenced the electromagnetic response even with the matrix type held constant.

In Figure 30, the vinylester matrix hybrids S11, S14, and S17 are compared. The carbon containing S11 and S14 exhibited low electromagnetic transmission and similar true absorption values (A_1_ = 18.93% and 19.11%, respectively), whereas S17 showed substantially higher transmission. Thus, carbon-containing vinylester hybrids provided comparable and superior electromagnetic absorption performance.

Among the epoxy matrix hybrid composites, S15 clearly stood out with an A_1_ value of 22.40% and a very low T_21_ value of 0.219% (Figure 31). S18 showed the second highest true absorption at 15.38%, whereas S12 remained at 5.82% because of its higher reflection. These results show that enhanced electromagnetic absorption is governed not only by the epoxy matrix but also by the fiber combination.

The results in Table 9 clearly demonstrate that total shielding effectiveness (SE_T_) and true absorption (A_1_) represent different performance metrics. The highest SE_T_ value was obtained for S10 (38.45 dB), followed by S2 (27.70 dB), S15 (26.60 dB), S3 (25.94 dB), and S1 (25.72 dB), whereas the highest A_1_ values were recorded for S15 (22.40%), S13 (19.18%), S14 (19.11%), S11 (18.93%), and S18 (15.38%). Thus, the strong shielding performance of the single carbon fiber composites was predominantly reflection driven, whereas the carbon/aramid hybrids showed a more balanced contribution of reflection and absorption. As emphasized by Hwang et al. [31], classification of a material as an electromagnetic “absorber” should consider not only SE_T_ or low S_21_ values but also the true absorption ratio referenced to the total incident electromagnetic energy. This interpretation is also consistent with the mechanisms discussed by Mikinka and Siwak [13] and the absorption oriented design principles of Isari et al. [37].

From an application perspective, the reduction in electromagnetic transmission to approximately 0.17–0.27% and SE_T_ values of 25.72–27.70 dB for S1–S3 indicate promising EMI shielding performance [7]. S10 likewise exhibited strong transmission suppression (T_21_ = 0.93%) and, with an SE_T_ of 38.45 dB, provided the highest total shielding effectiveness in the present dataset. However, their predominantly reflection driven behavior may be a limitation where back reflection is undesirable [2,8]. In contrast, S15 combined a very low transmission ratio of 0.21% with a true absorption ratio of 22.40%, making it particularly noteworthy for reducing reflection while dissipating electromagnetic energy within the material. These characteristics support the potential of carbon/aramid–epoxy hybrids for lightweight defense, aerospace, space, and automotive structures requiring both structural functionality and EMI shielding. Nevertheless, classification as radar absorbing or low observable materials would require additional evaluation of reflection loss, complex permittivity and permeability, impedance matching, thickness, and angle of incidence effects.

The standard deviation analysis of the S-parameters demonstrated good repeatability of the electromagnetic measurements over the 4–40 GHz frequency range (Table 10). Across all 18 configurations, the mean SD values calculated from S_11_, S_21_, S_12_, and S_22_ ranged from 0.06 to 0.1 dB, with the maximum value remaining below 0.14 dB. Carbon-rich configurations generally exhibited slightly higher dispersion in the transmission parameters (S_21_ and S_12_) than in the reflection parameters (S_11_ and S_22_); however, these variations were small relative to the pronounced differences in electromagnetic performance among the configurations.

As shown in Table 10, S10, which exhibited the highest total shielding effectiveness (38.45 dB) with very low transmission (~0.93%), showed a mean SD of only 0.07 dB. Similarly, the carbon/aramid epoxy configuration S15, characterized by the highest incident-power absorption ratio (22.40%) and extremely low transmission (~0.22%), exhibited a mean SD of 0.09 dB. The lowest mean SD values were obtained for S16 (0.06 dB), S17 (0.06 dB), and S18 (0.06 dB). Overall, the consistently low SD values reported in Table 10 support the repeatability of the measurements and indicate that the observed differences in shielding, transmission, reflection, and absorption behavior are predominantly associated with the fiber/matrix combination and hybrid architecture rather than experimental variability.

## 4. Conclusions

The comparative evaluation of 18 Coremat interlayered composite configurations demonstrated that their mechanical performance was jointly governed by fiber type, matrix type, and hybrid architecture. The carbon/carbon epoxy specimen S3 exhibited the highest tensile strength (231.2 MPa), with a mean tensile strength of 231.2 ± 16.6 MPa, demonstrating the effectiveness of the carbon fiber epoxy combination when maximum tensile load bearing capacity is the primary requirement. Among the hybrid systems, the carbon/aramid epoxy specimen S15 provided the most balanced tensile response by combining a tensile strength of 212.9 MPa with the highest maximum strain of 0.10 mm/mm. Its mean tensile strength of 212.9 ± 15.8 MPa further showed that this high tensile performance was maintained when specimen-to-specimen variability was considered. In flexural loading, the glass/glass–vinylester specimen S5 achieved the highest flexural strength (160.5 MPa), whereas aramid-containing specimens, particularly S7, S15, and S16, exhibited enhanced deformation capacity. These results show that hybridization did not necessarily increase maximum tensile or flexural strength but modified the balance among strength, deformation capacity, and damage tolerance. The mean ± SD analysis, together with the configuration-level statistical comparisons, further demonstrated that the observed mechanical differences were systematic and not attributable solely to within-configuration experimental dispersion.

The thermomechanical results revealed a pronounced dependence on the specific fiber–matrix combination. The glass/glass vinylester specimen S5 exhibited both the highest heat deflection temperature (128.0 °C) and the highest Vicat softening temperature (132.7 °C). Among the hybrid configurations, the carbon/aramid vinylester specimen S14 reached the highest heat deflection temperature (80.7 °C), while the carbon/aramid–epoxy specimen S15 achieved a Vicat softening temperature of 131.5 °C. In contrast, the Vicat softening temperatures of the glass/aramid hybrid specimens remained within a narrow range of 67.9–72.0 °C, indicating a limited matrix effect within this architecture. Consideration of the experimental dispersion supported the reproducibility of the thermomechanical trends observed among the investigated configurations.

The electromagnetic measurements demonstrated that the single carbon specimens S1–S3 provided very low transmission and high total shielding effectiveness, although their shielding response was predominantly reflection driven. The carbon/glass–polyester hybrid S10 exhibited the highest total shielding effectiveness of 38.45 dB, whereas the carbon/aramid epoxy specimen S15 achieved the highest true absorption ratio of 22.40%, together with a very low transmission ratio of 0.22%.

Across all 18 configurations, the mean standard deviation of the S-parameters remained below 0.10 dB (0.06–0.1 dB), demonstrating high measurement repeatability over the 4–40 GHz frequency range. Notably, S10 achieved the highest total shielding effectiveness (38.45 dB) with a mean SD of only 0.07 dB, indicating that its superior shielding response was substantially larger than the associated measurement dispersion.

Therefore, total shielding effectiveness and true absorption should be considered as distinct performance criteria, and the preferred configuration should be selected according to the required balance among mechanical, thermomechanical, and electromagnetic properties. Taken together, the mechanical, thermomechanical, and electromagnetic dispersion analyses indicate that the principal performance trends observed among the investigated configurations were reproducible within the experimental variability of the measurements. Within the specific fiber areal weights, matrix systems, and laminate architectures investigated in this study, the results indicate the potential of carbon-containing configurations for lightweight electromagnetic shielding applications where low electromagnetic transmission is desirable. These findings should, however, be regarded as a preliminary indication of application potential rather than direct evidence of in-service performance in defense, aerospace, space, automotive, or electric transportation systems. Further validation, including impact, fatigue, flammability, environmental aging, moisture absorption, permittivity, permeability, and angular response testing, would be required before application specific performance can be established. Moreover, the comparative trends reported here are specific to the materials, fiber areal weights, manufacturing conditions, and laminate architectures examined in this study and should not be interpreted as a universal ranking of carbon, glass, and aramid fibers or polyester, vinylester, and epoxy matrices. The S15 carbon/aramid epoxy configuration is particularly promising for multifunctional structures requiring high tensile performance together with reduced reflection, increased electromagnetic energy dissipation, and very low transmission.

## Figures and Tables

**Figure 1:**
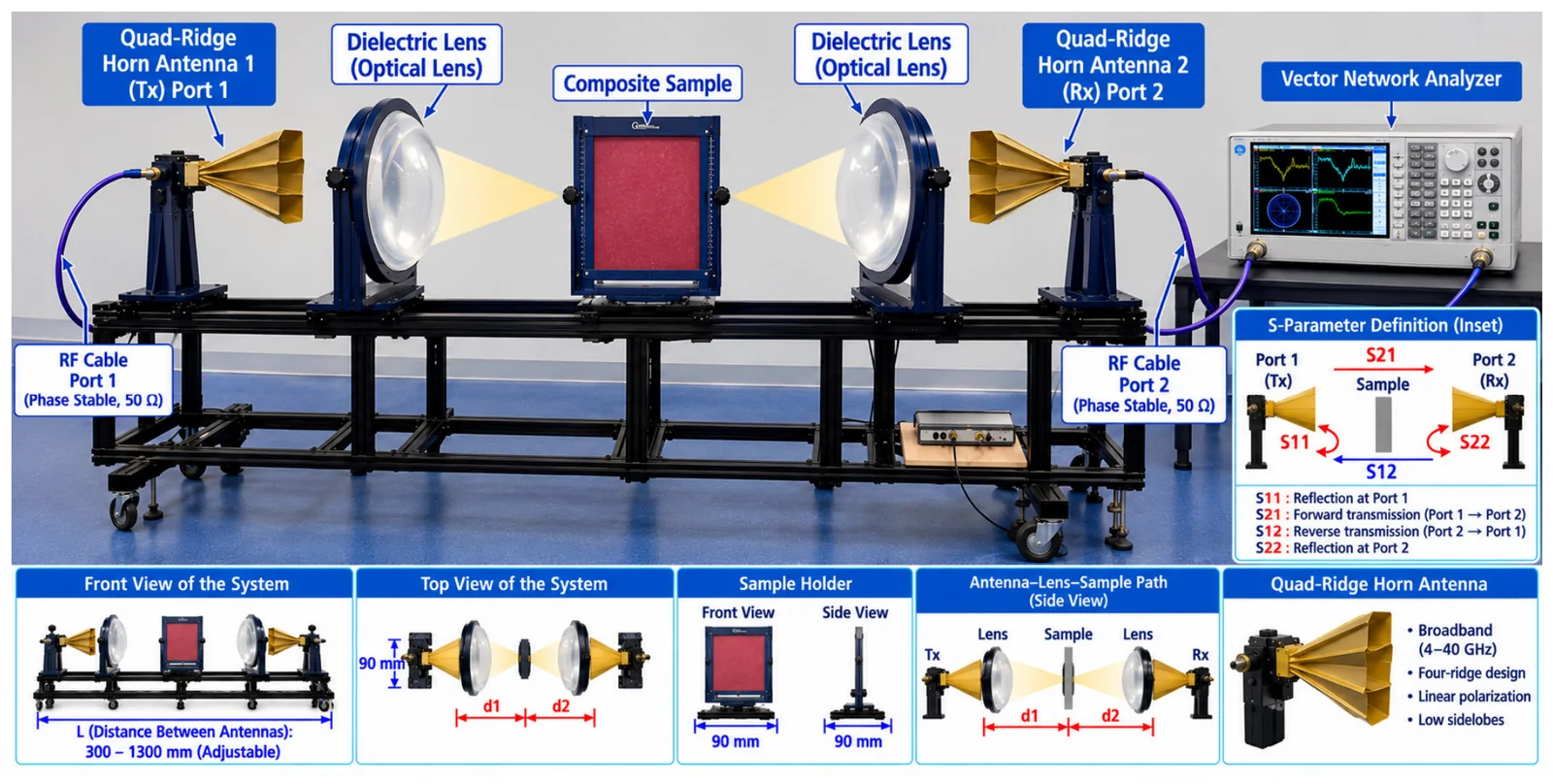
Free space electromagnetic measurement setup using a Rohde & Schwarz VNA and horn antennas.

**Figure 2:**
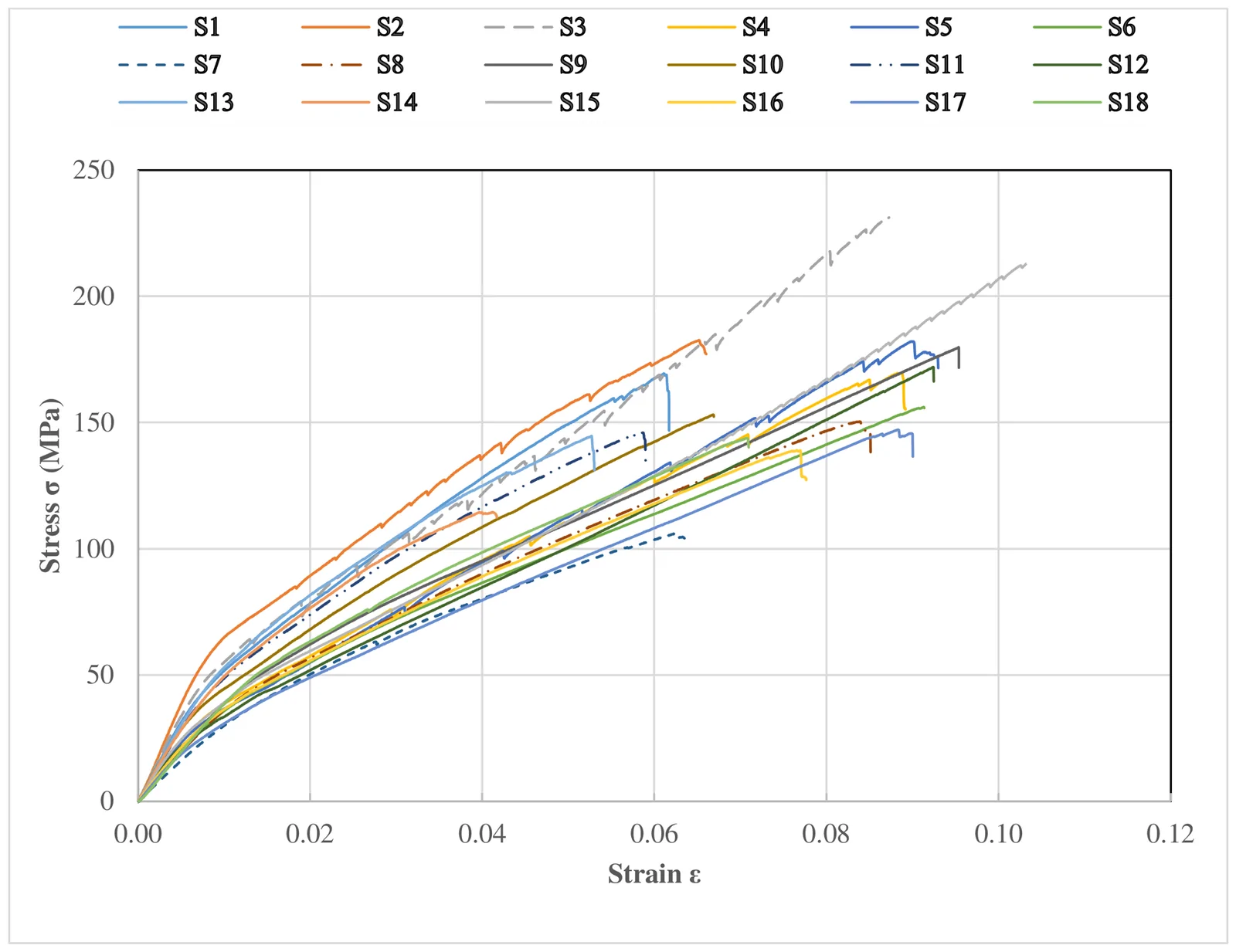
Tensile test stress–strain curves.

**Figure 3:**
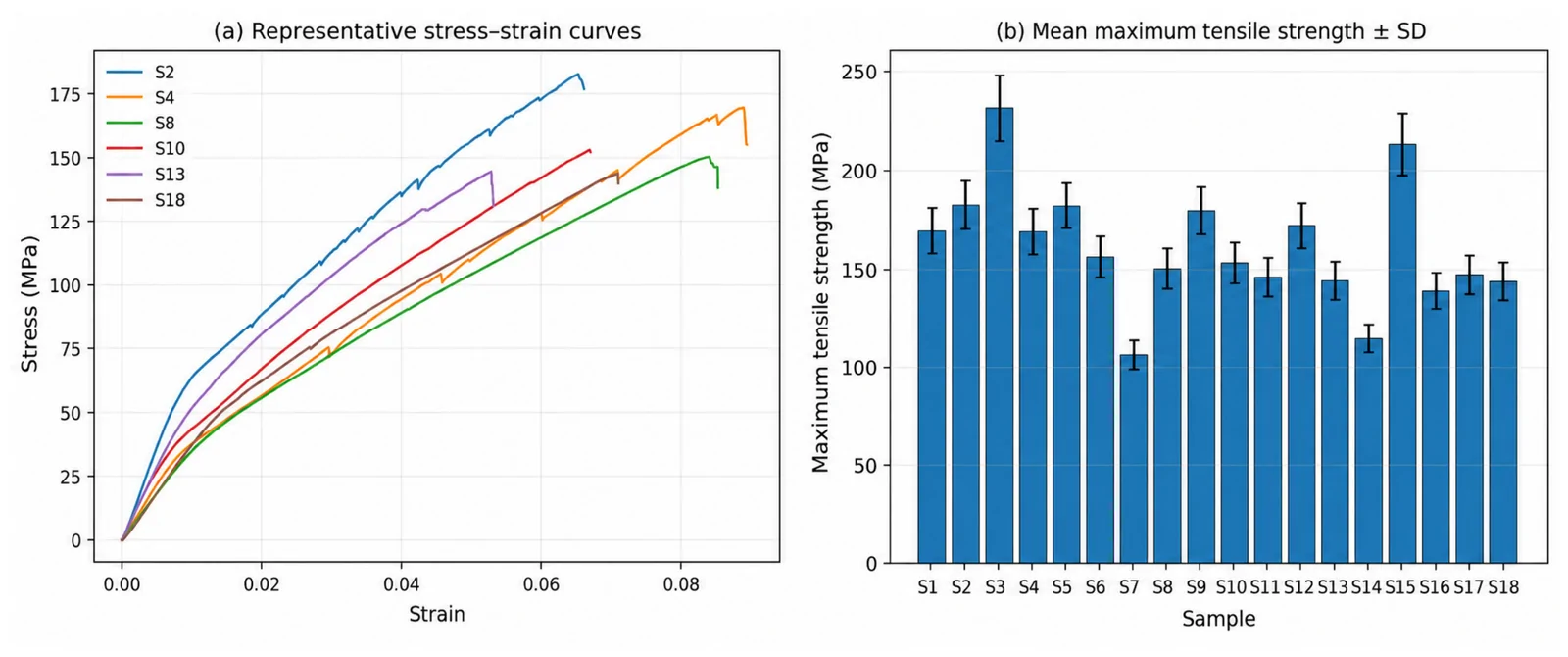
Tensile behavior and statistical distribution of the investigated composite configurations: (**a**) representative stress–strain curves selected from each configuration group to illustrate the characteristic tensile response and (**b**) maximum tensile strength of S1–S18 expressed as mean ± standard deviation (SD, n = 5). Error bars represent the standard deviation of five specimens tested for each configuration.

**Figure 4:**
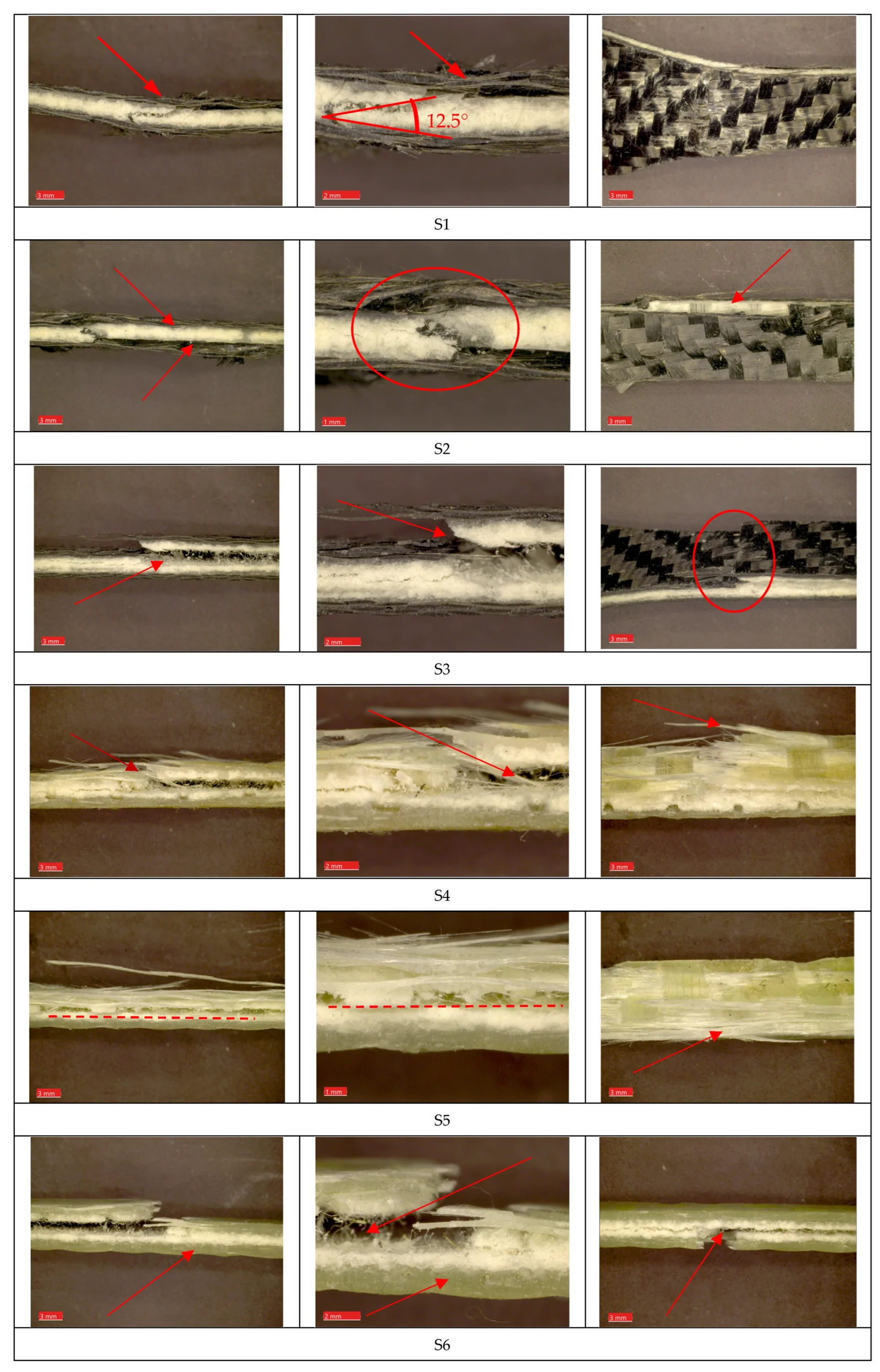
Macroscopic cross-sectional views of the fracture regions (S1–S6).

**Figure 5:**
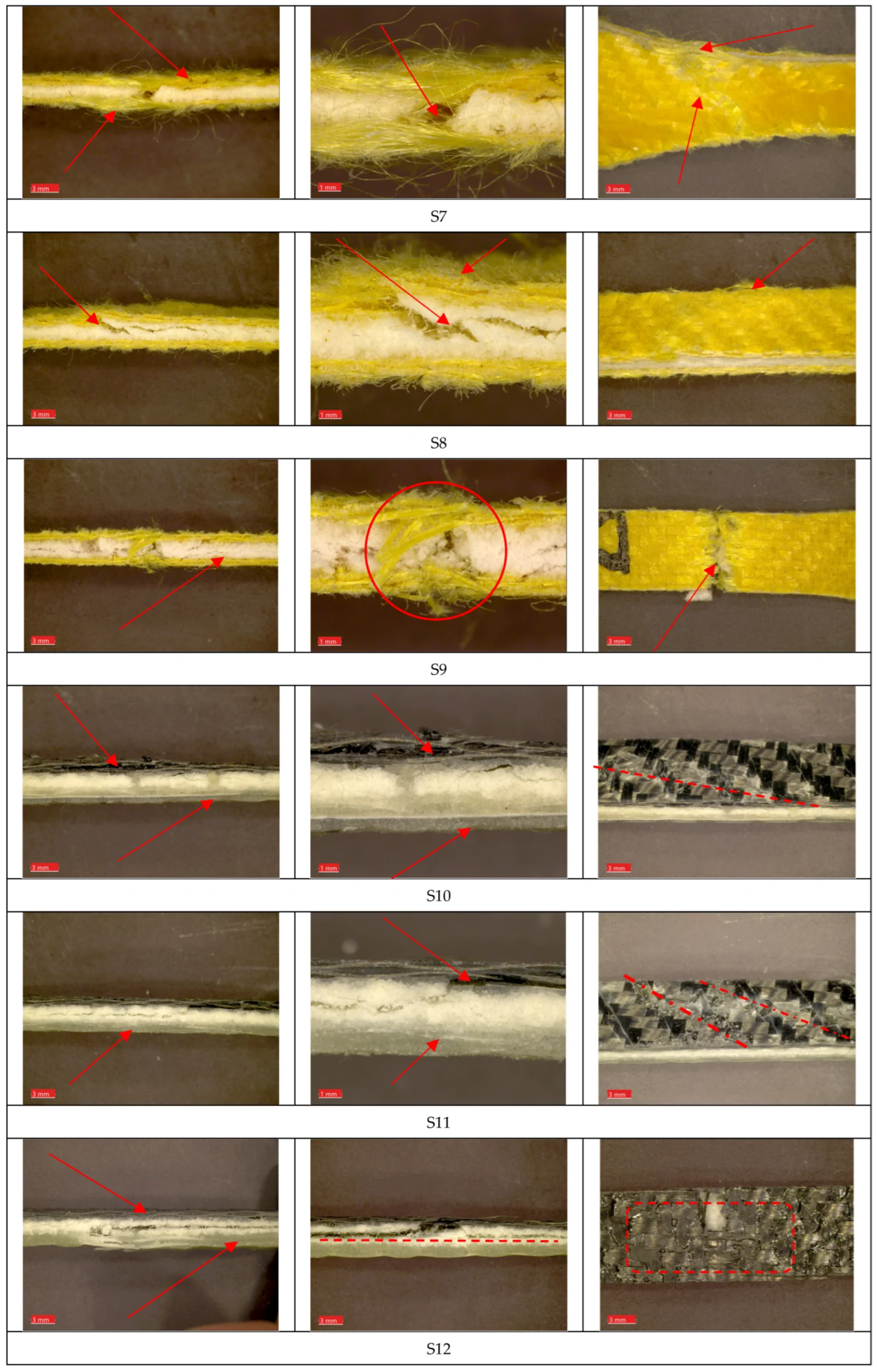
Macroscopic cross-sectional views of the fracture regions (S7–S12).

**Figure 6:**
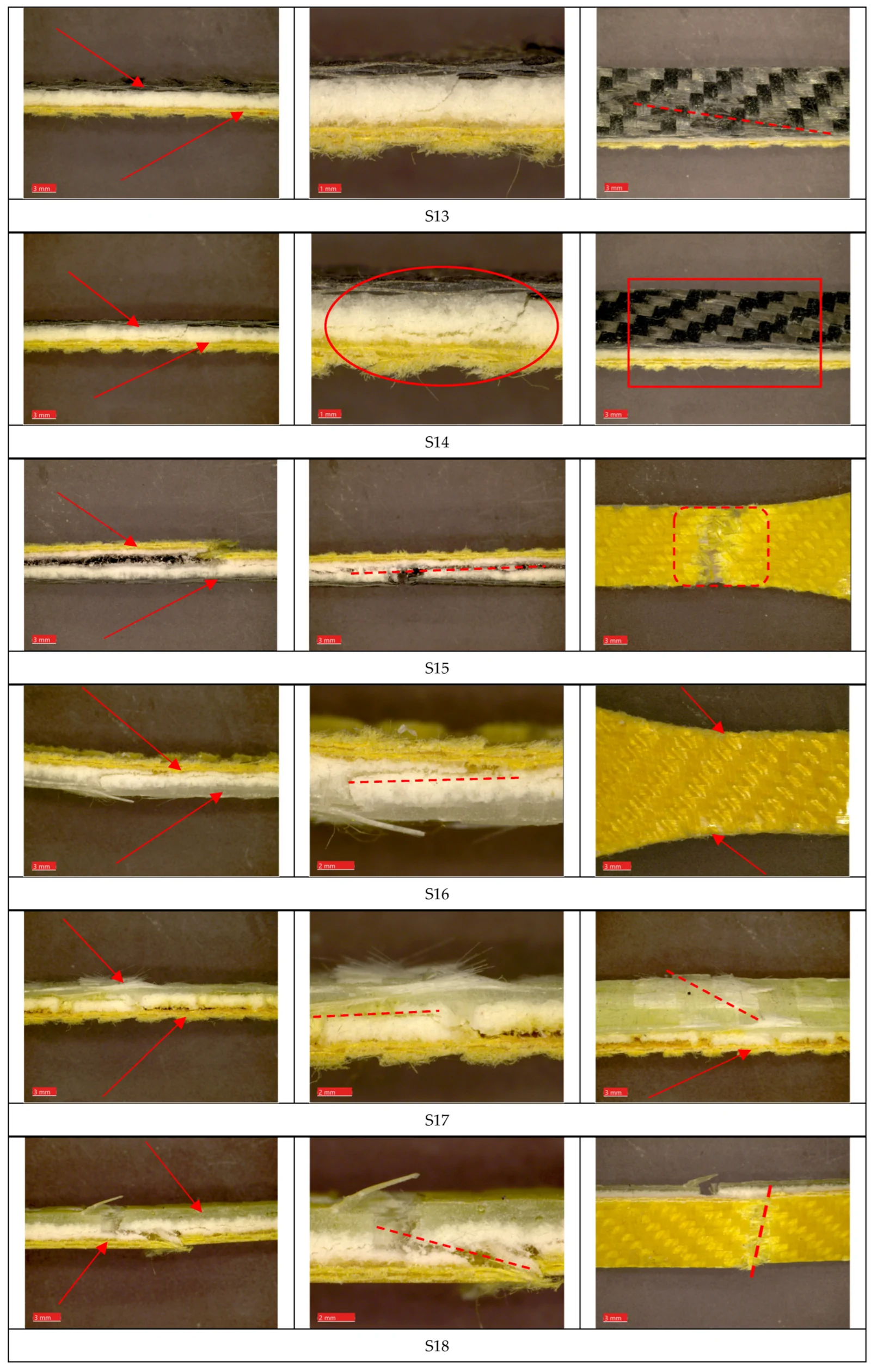
Macroscopic cross-sectional views of the fracture regions (S13–S18).

**Figure 7:**
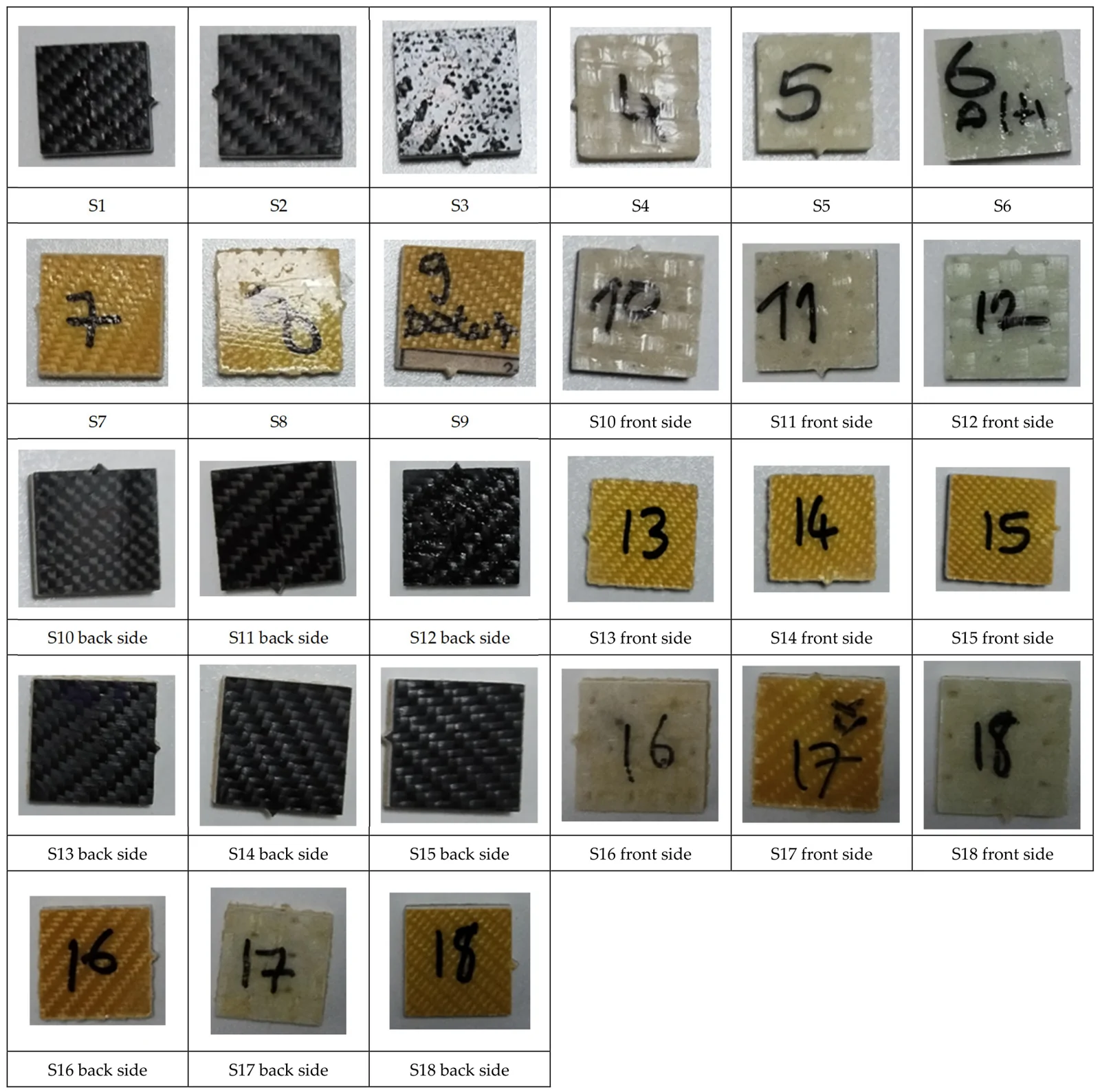
Vicat deformed specimens.

**Figure 8:**
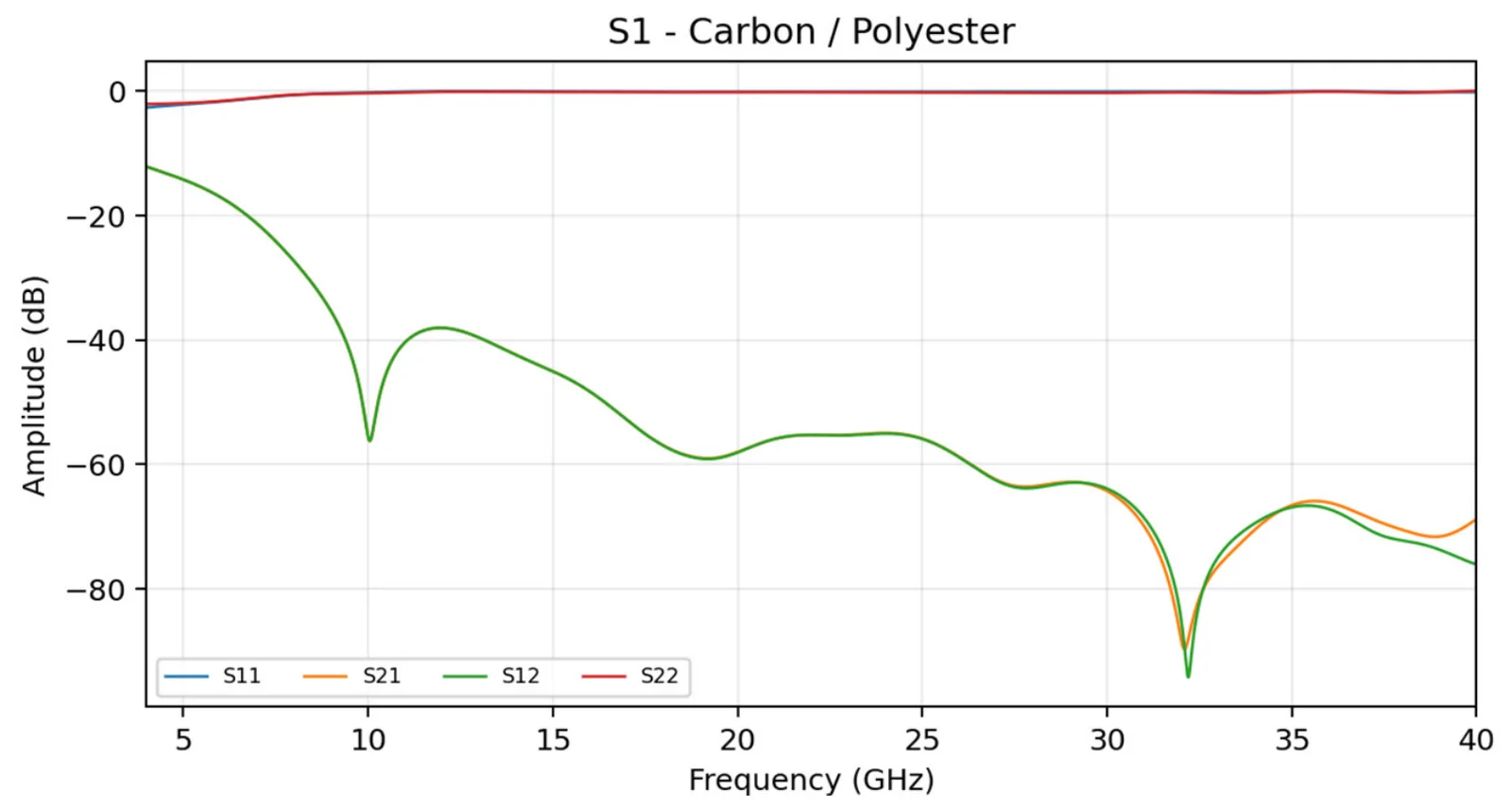
S_11_, S_21_, S_12_, and S_22_ curves of the S1 (carbon/polyester) specimen over the 4–40 GHz frequency range.

**Figure 9:**
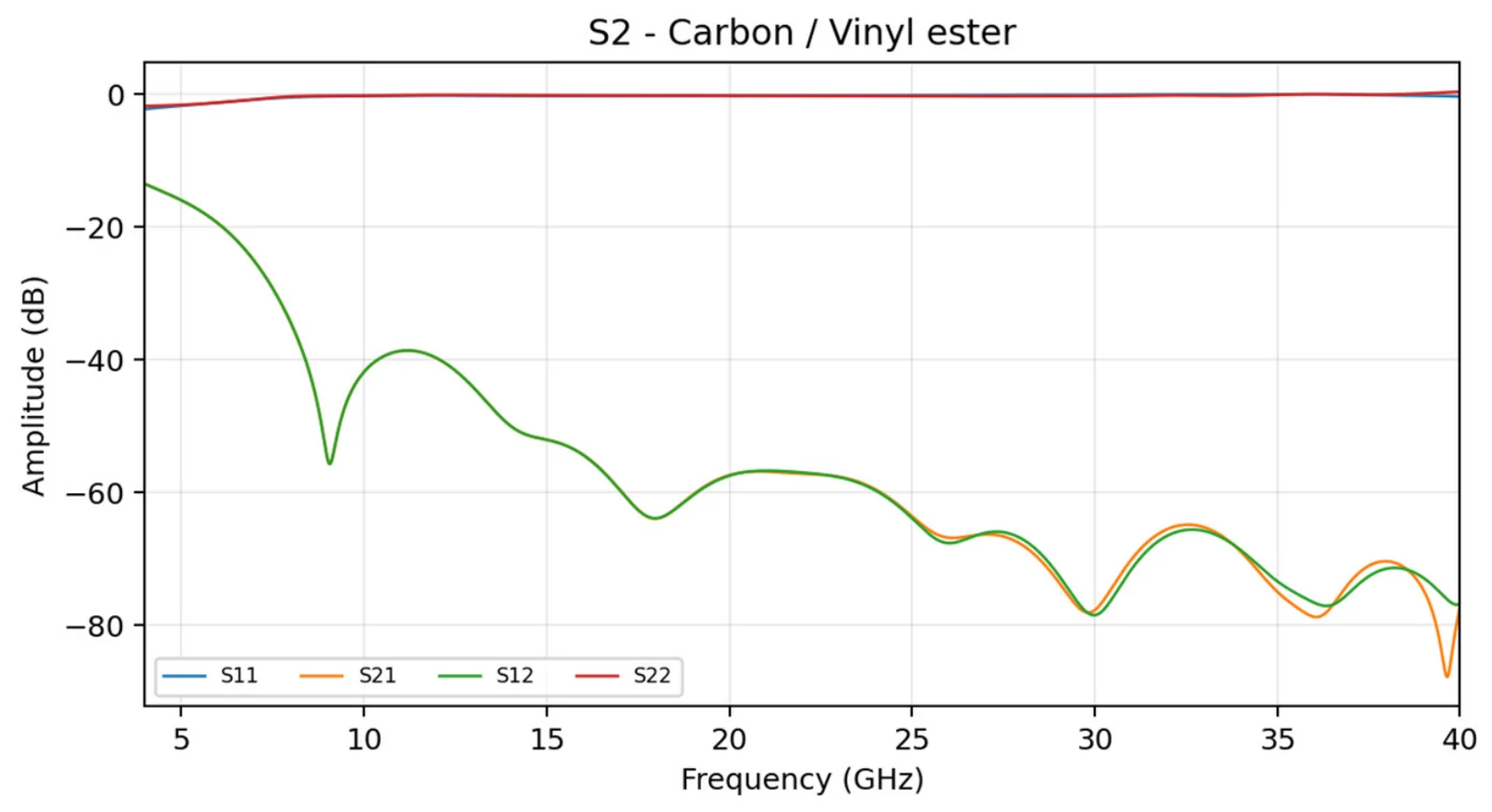
S_11_, S_21_, S_12_, and S_22_ curves of the S2 (carbon/vinylester) specimen over the 4–40 GHz frequency range.

**Figure 10:**
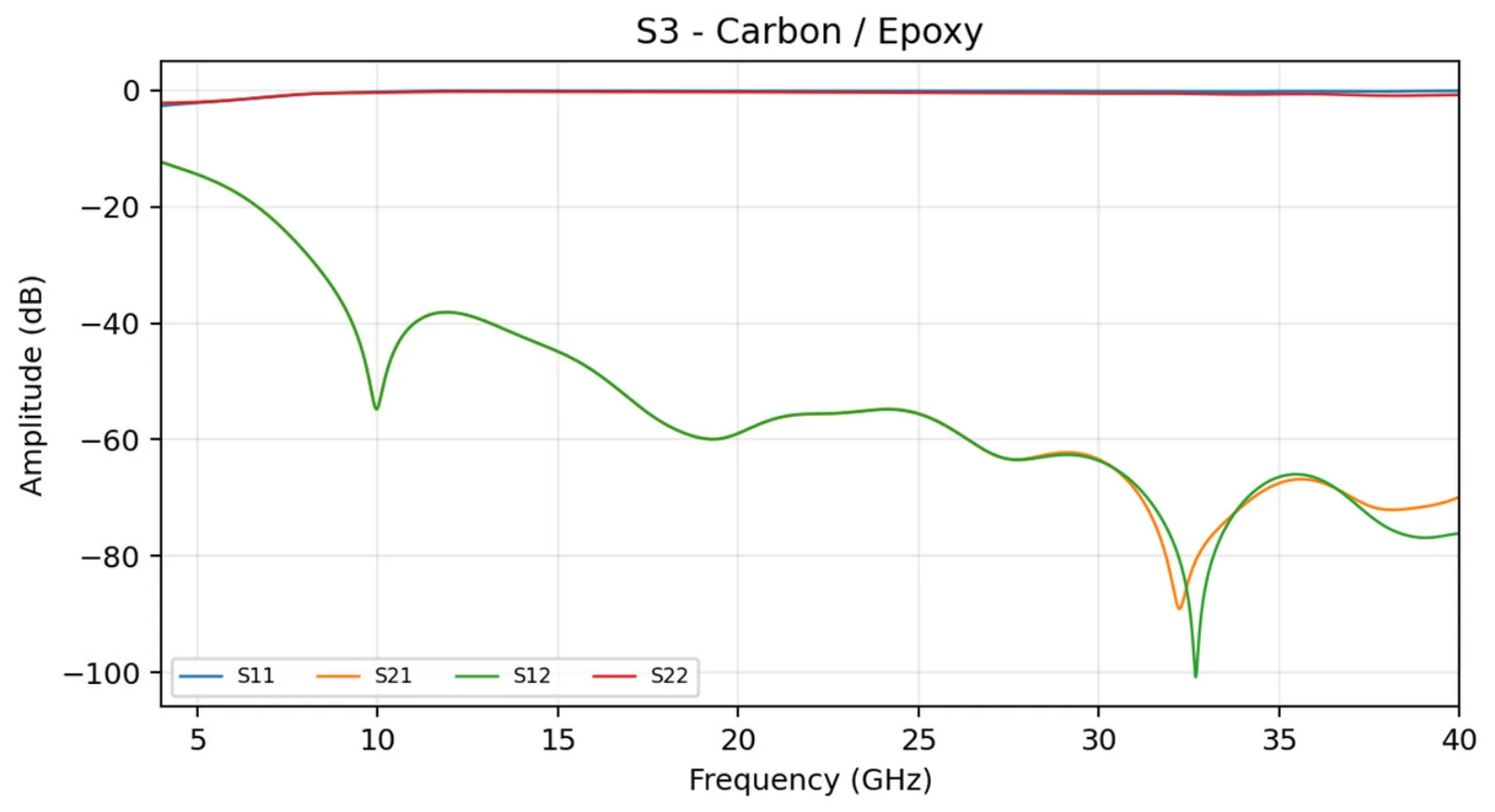
S_11_, S_21_, S_12_, and S_22_ curves of the S3 (carbon/epoxy) specimen over the 4–40 GHz frequency range.

**Figure 11:**
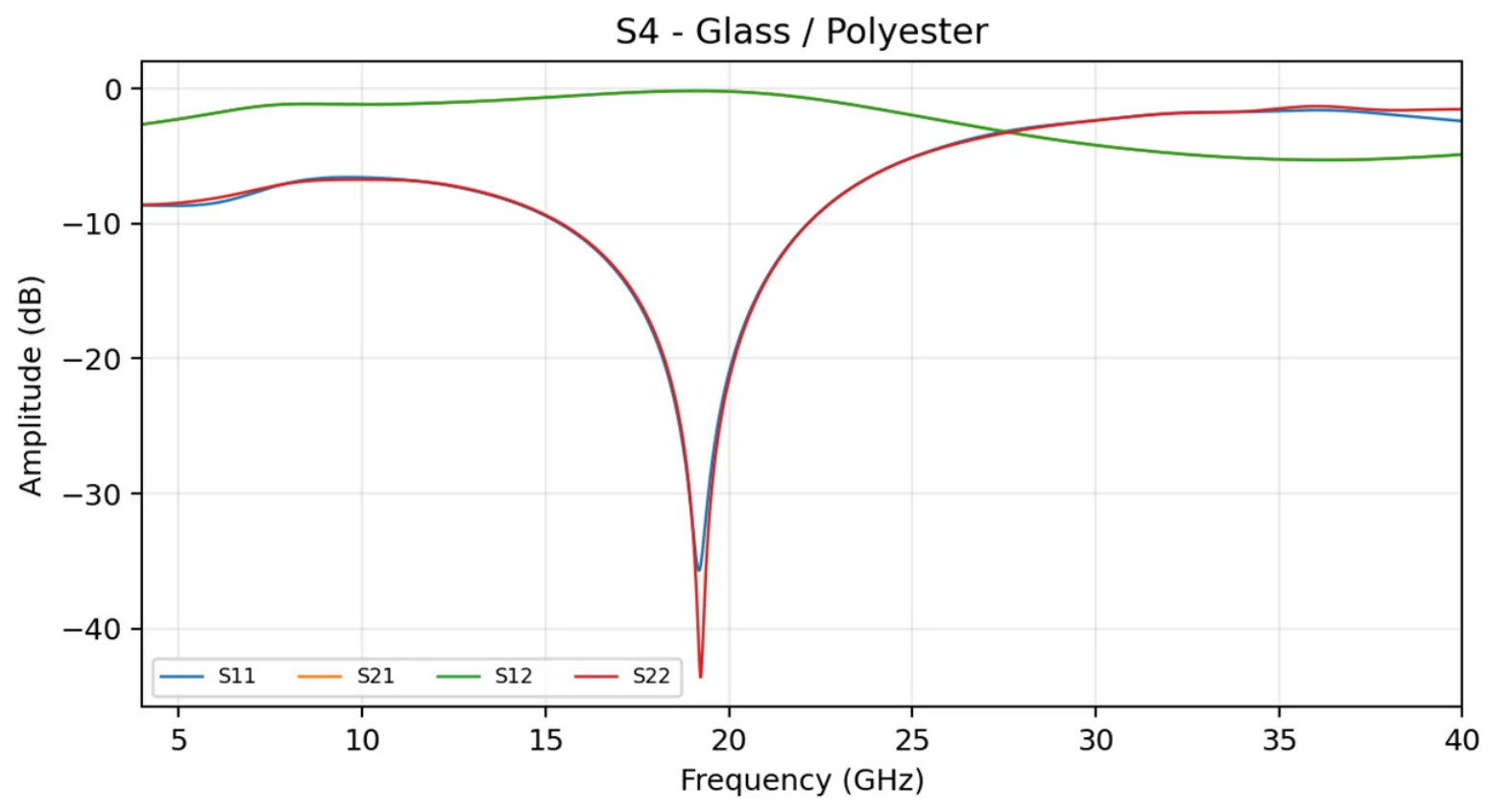
S_11_, S_21_, S_12_, and S_22_ curves of the S4 (glass/polyester) specimen over the 4–40 GHz frequency range.

**Figure 12:**
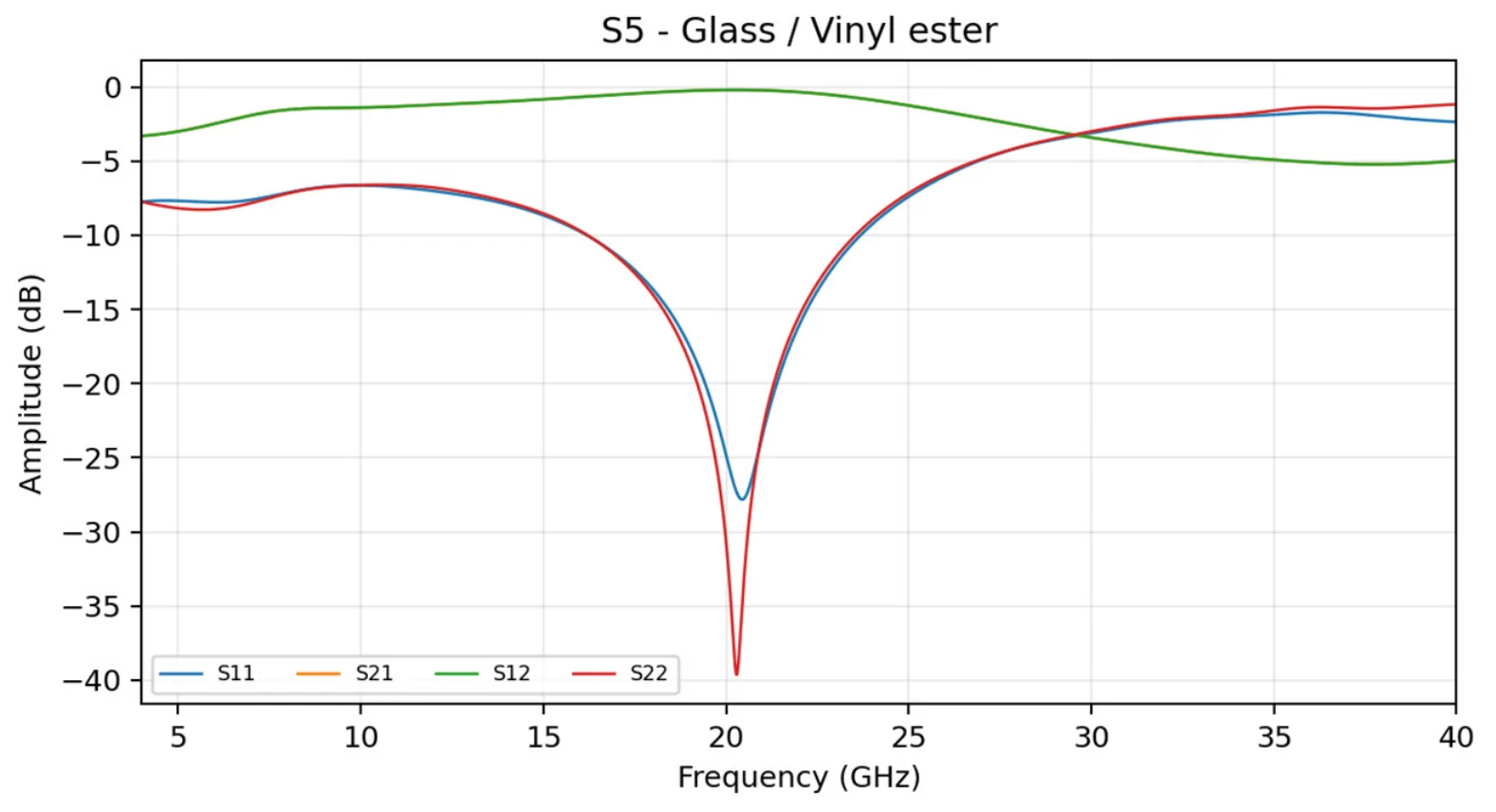
S_11_, S_21_, S_12_, and S_22_ curves of the S5 (glass/vinylester) specimen over the 4–40 GHz frequency range.

**Figure 13:**
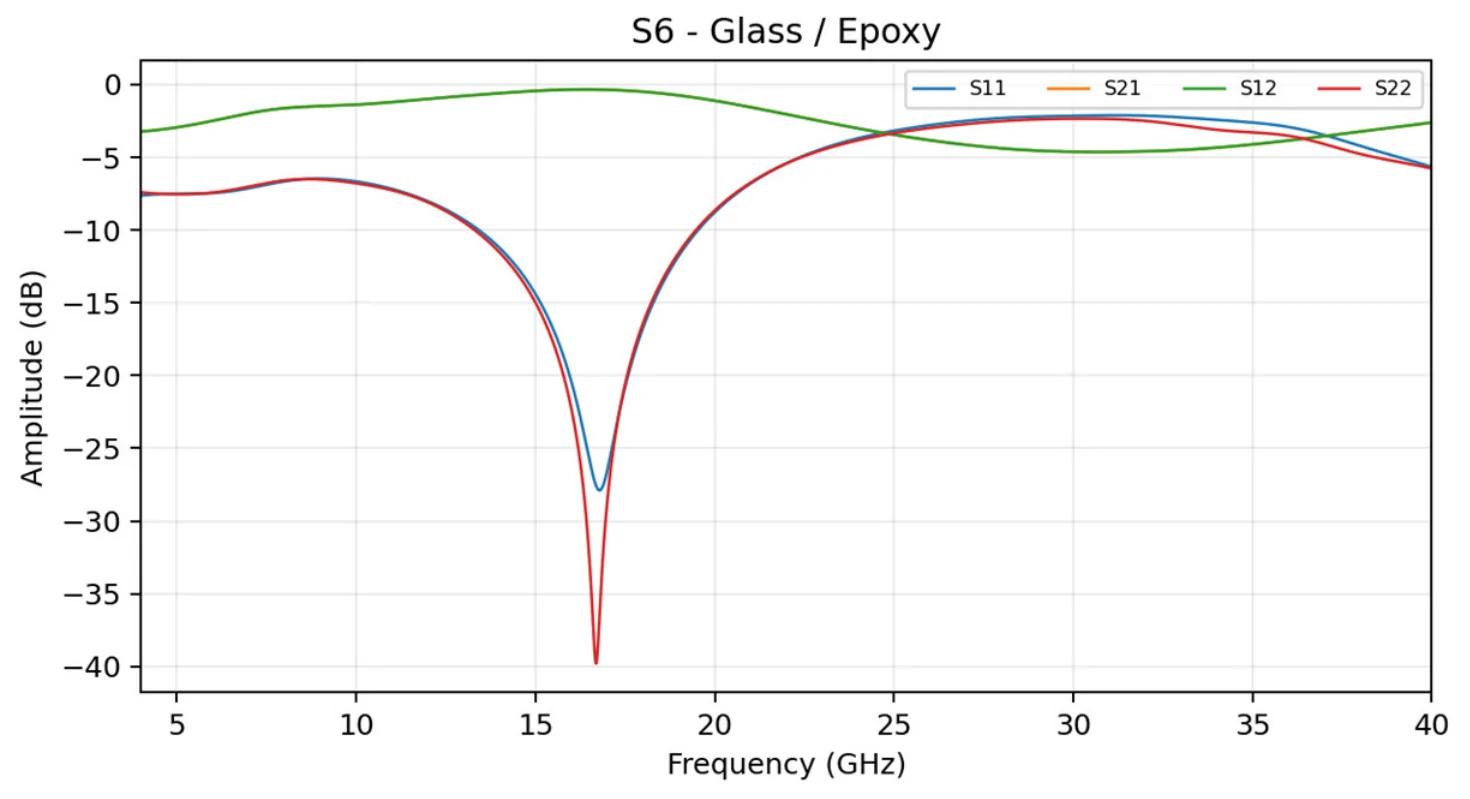
S_11_, S_21_, S_12_, and S_22_ curves of the S6 (glass/epoxy) specimen over the 4–40 GHz frequency range.

**Figure 14:**
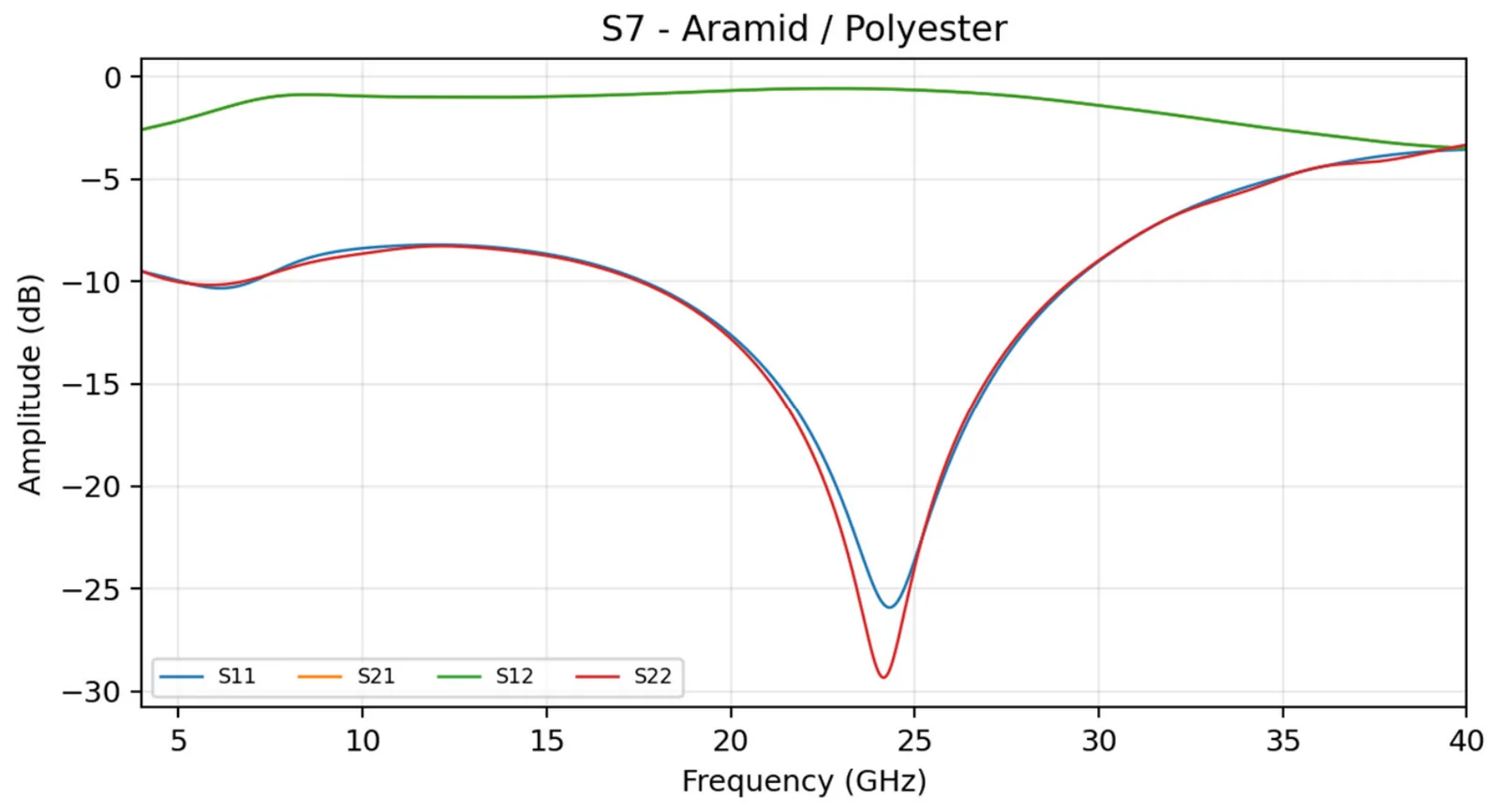
S_11_, S_21_, S_12_, and S_22_ curves of the S7 (aramid/polyester) specimen over the 4–40 GHz frequency range.

**Figure 15:**
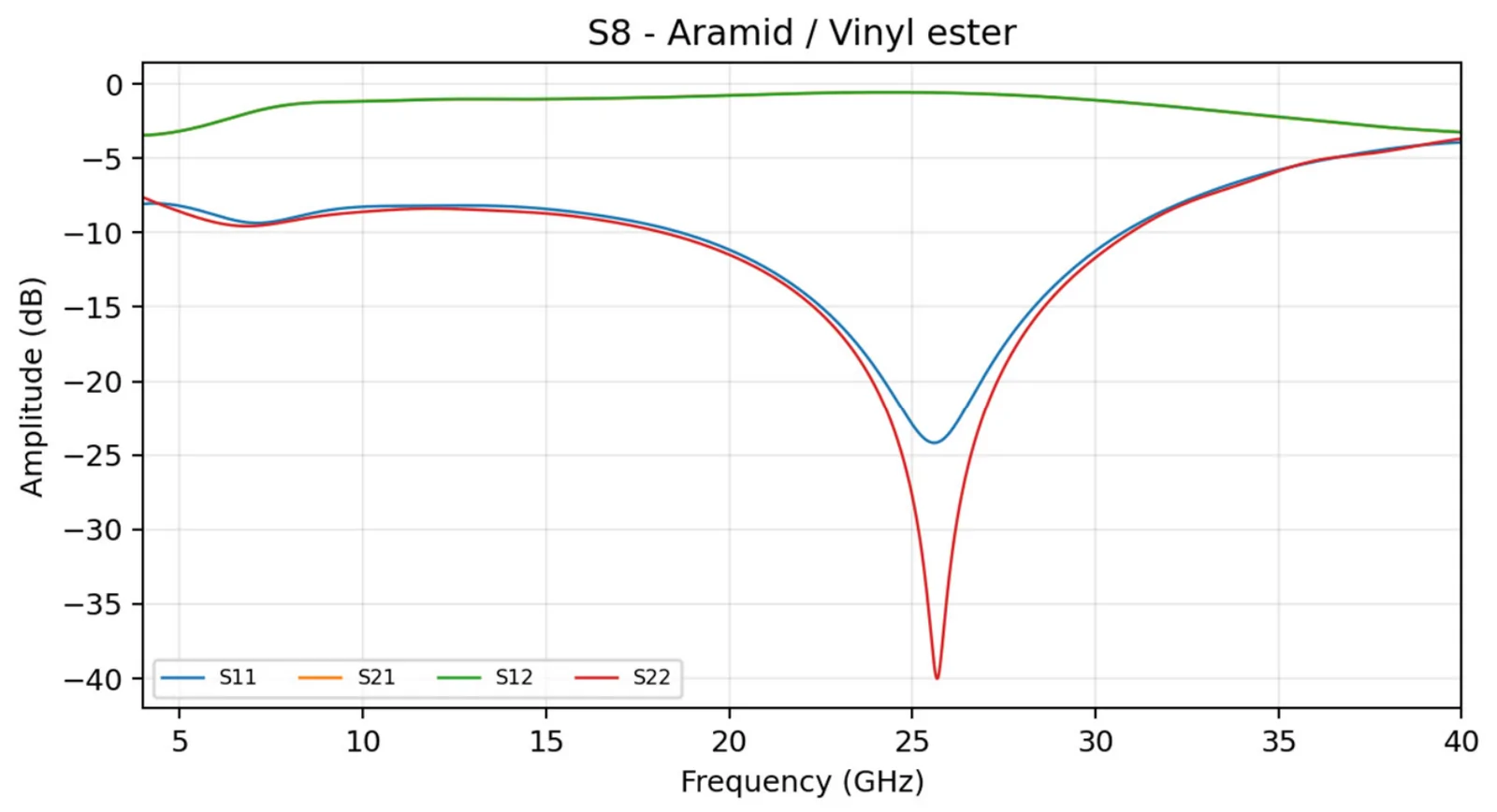
S_11_, S_21_, S_12_, and S_22_ curves of the S8 (aramid/vinylester) specimen over the 4–40 GHz frequency range.

**Figure 16:**
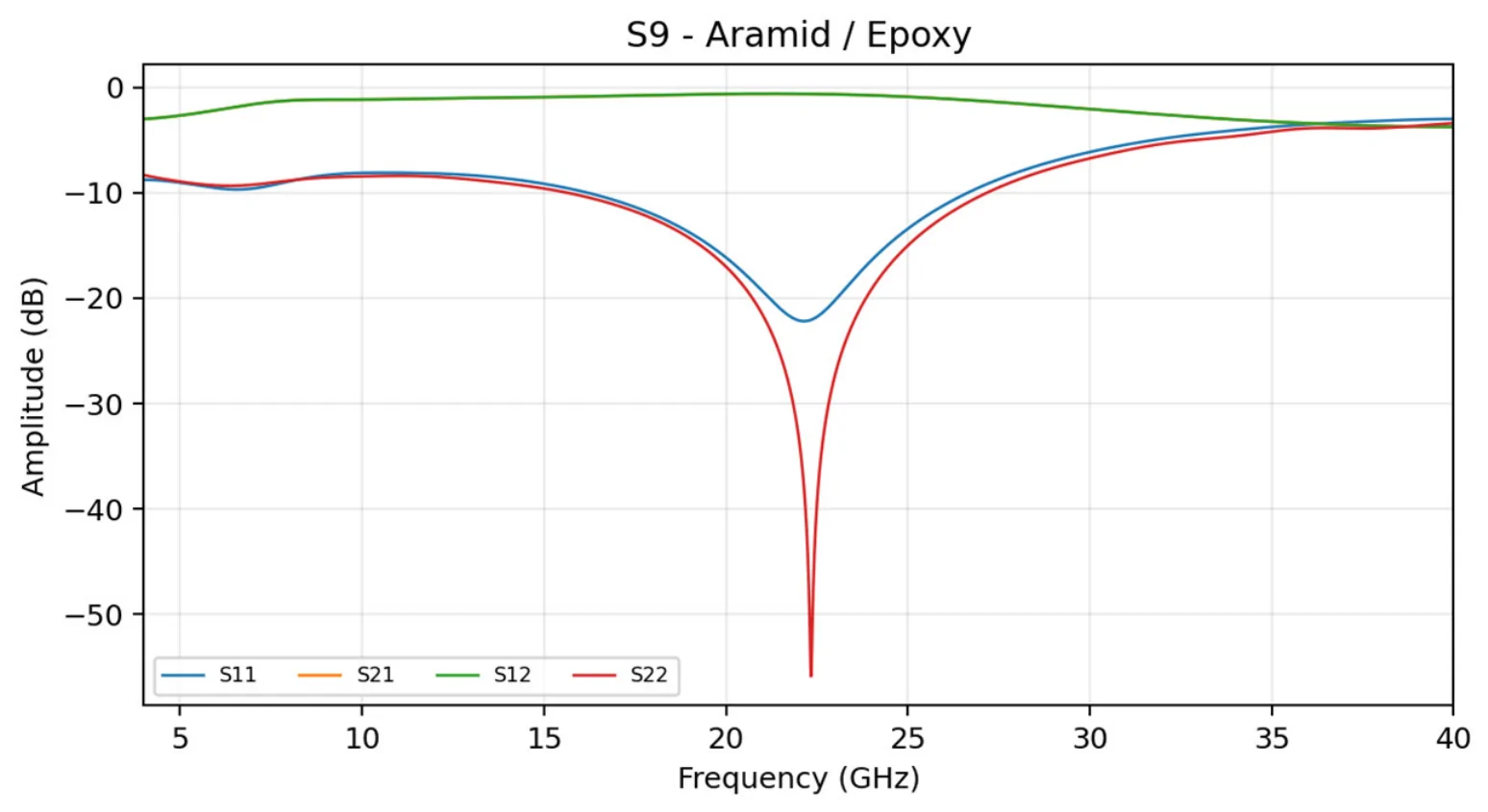
S_11_, S_21_, S_12_, and S_22_ curves of the S9 (aramid/epoxy) specimen over the 4–40 GHz frequency range.

**Figure 17:**
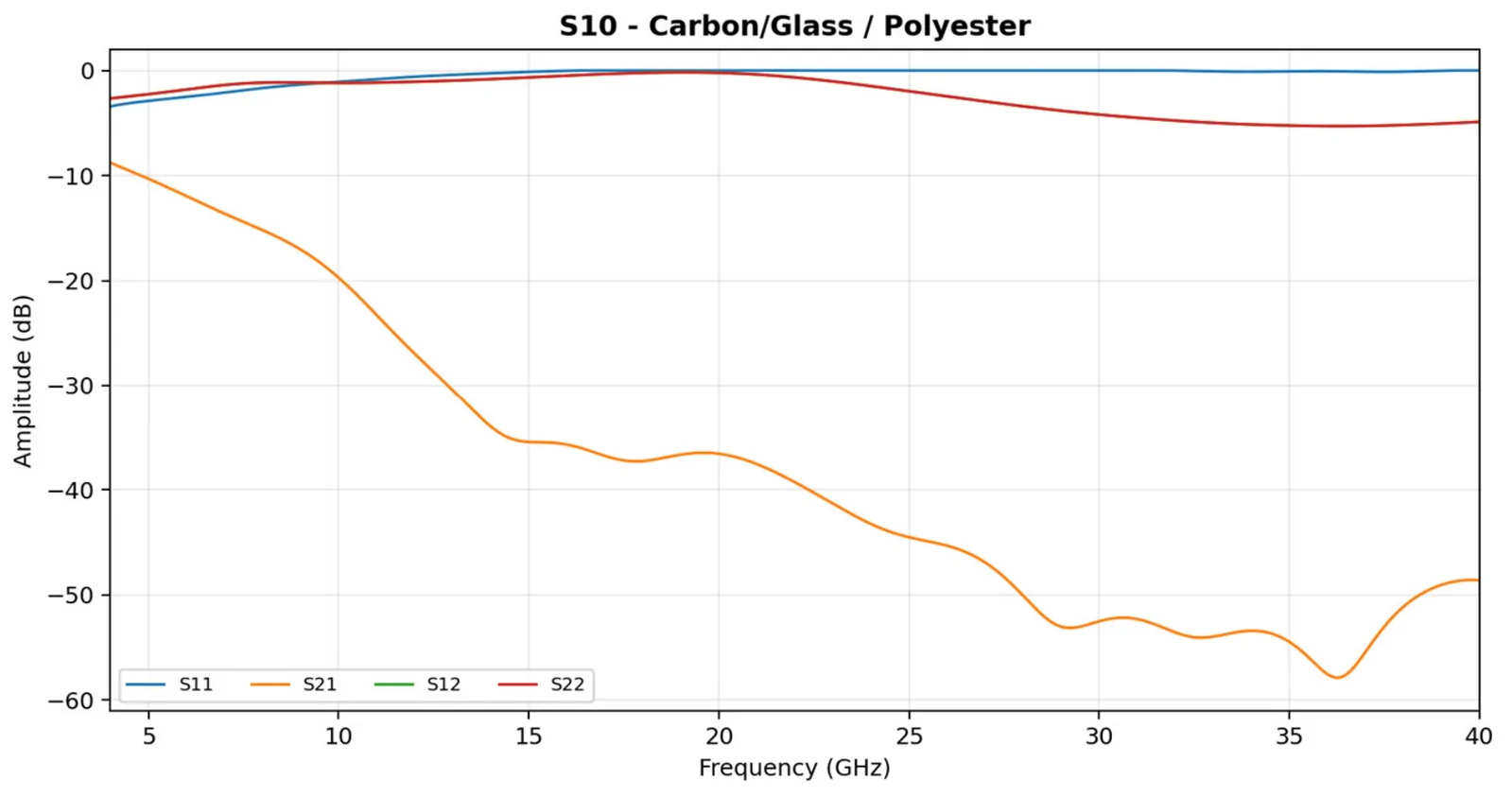
S_11_, S_21_, S_12_, and S_22_ curves of the S10 (carbon/glass/polyester) specimen over the 4–40 GHz frequency range.

**Figure 18:**
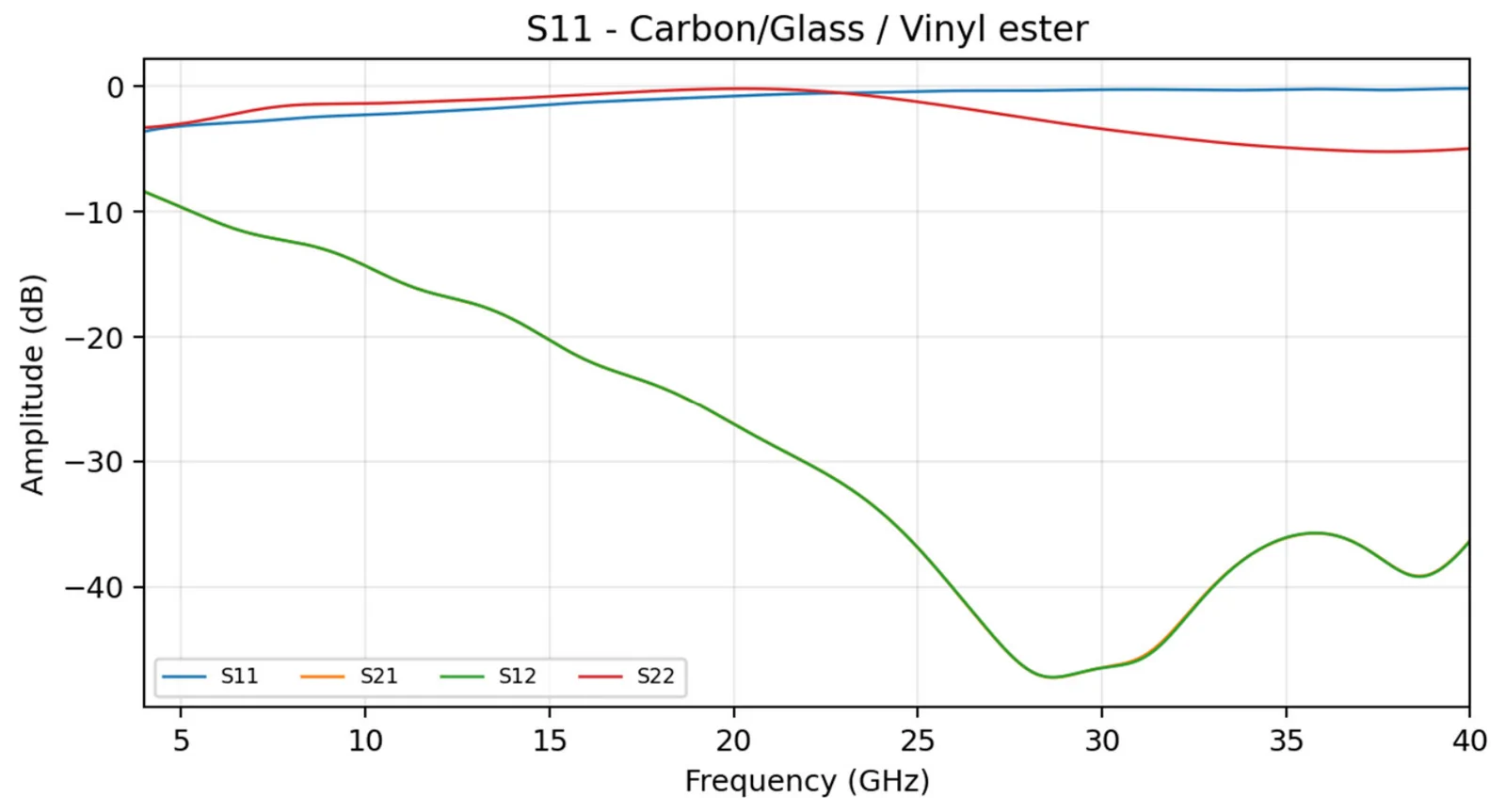
S_11_, S_21_, S_12_, and S_22_ curves of the S11 (carbon/glass/vinylester) specimen over the 4–40 GHz frequency range.

**Figure 19:**
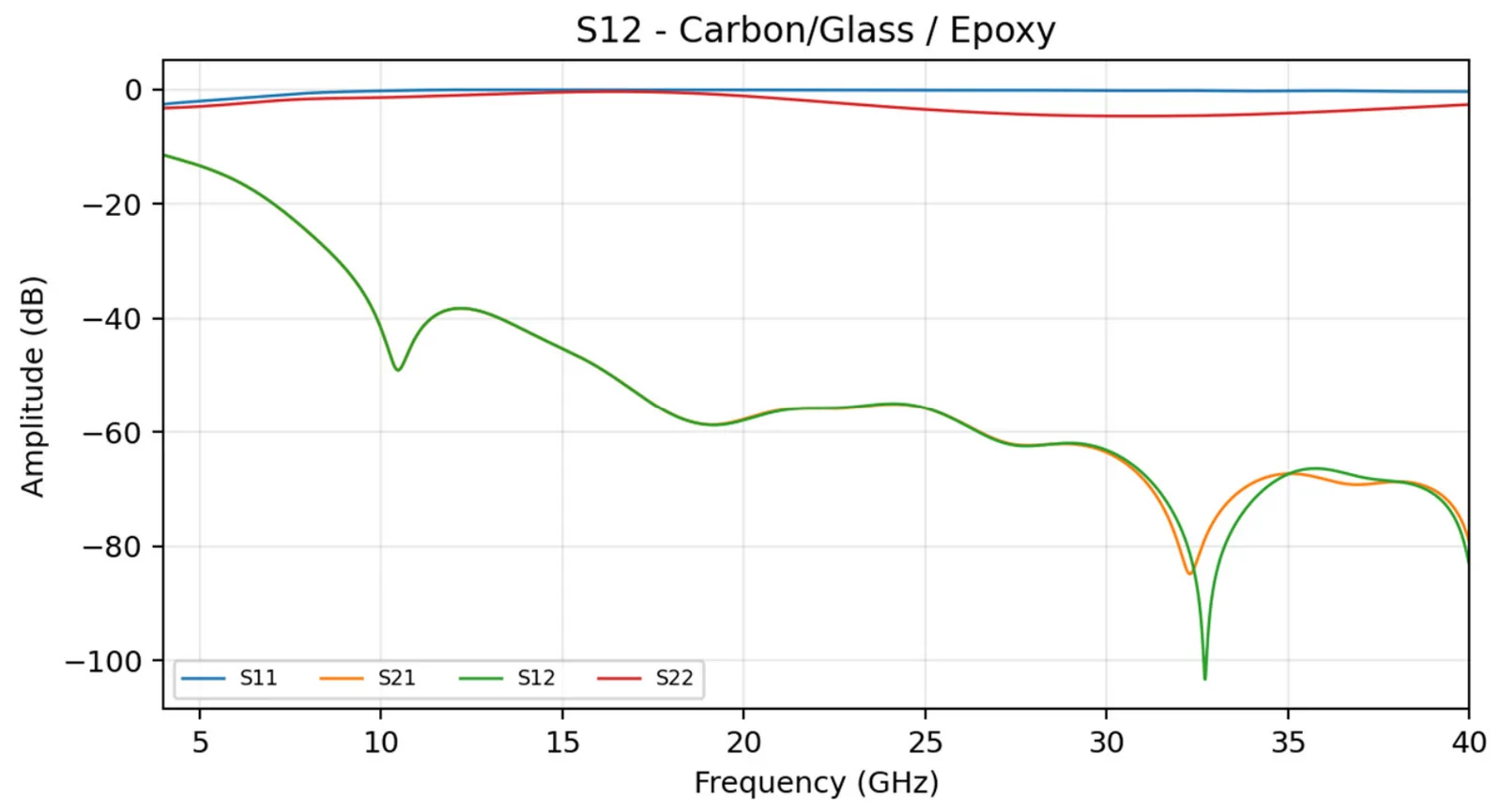
S_11_, S_21_, S_12_, and S_22_ curves of the S12 (carbon/glass/epoxy) specimen over the 4–40 GHz frequency range.

**Figure 20:**
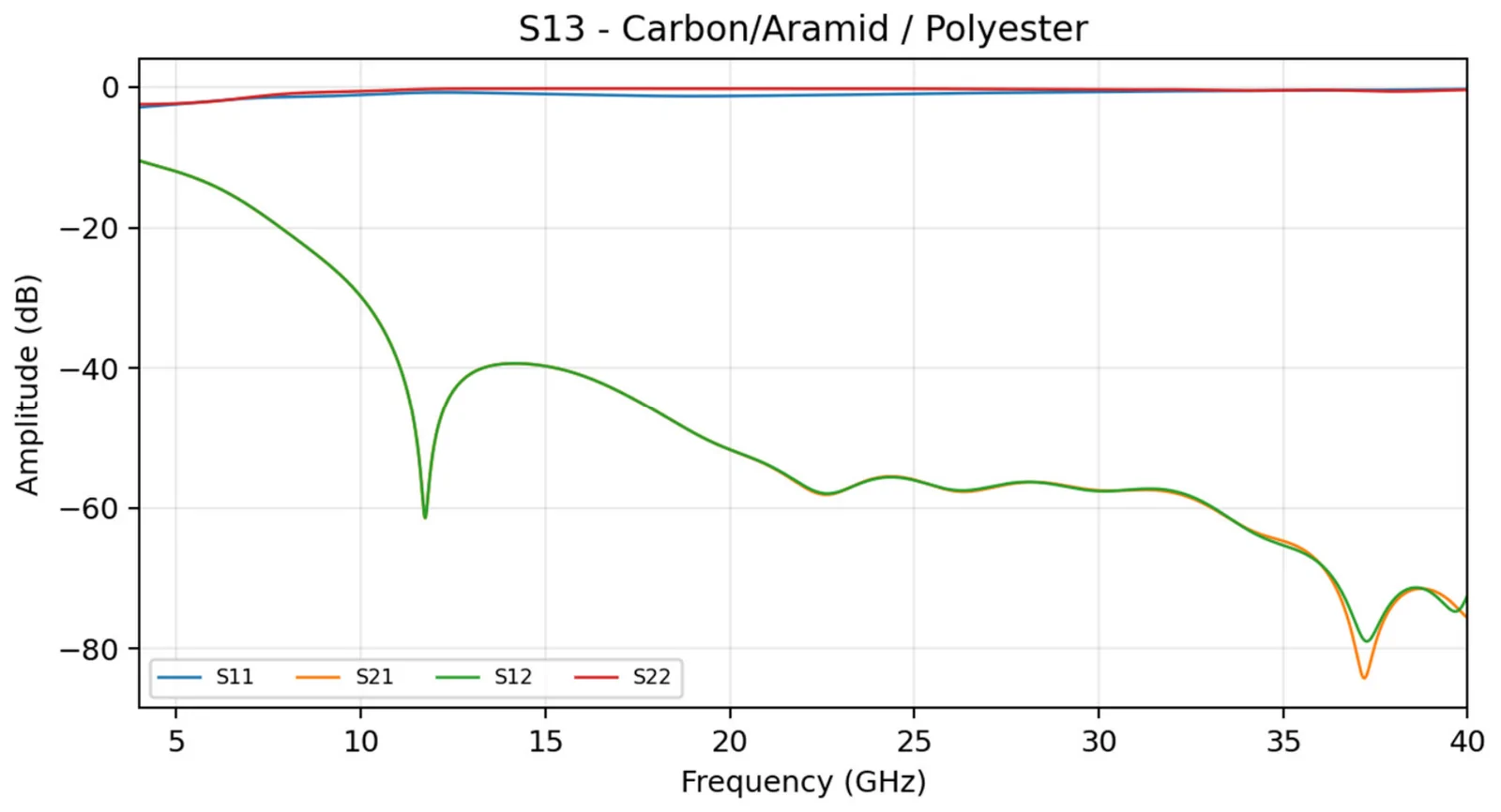
S_11_, S_21_, S_12_, and S_22_ curves of the S13 (carbon/aramid/polyester) specimen over the 4–40 GHz frequency range.

**Figure 21:**
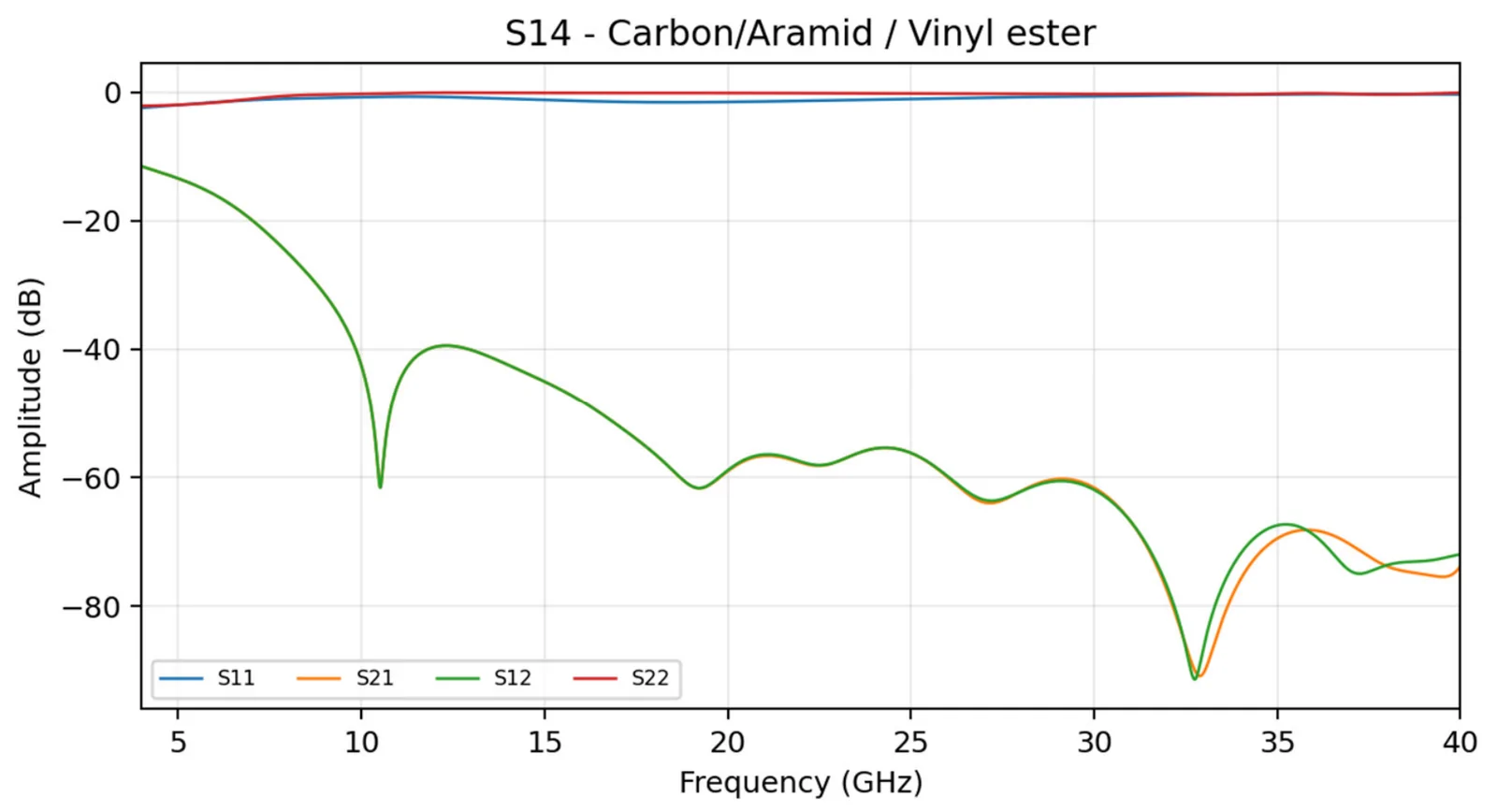
S_11_, S_21_, S_12_, and S_22_ curves of the S14 (carbon/aramid/vinylester) specimen over the 4–40 GHz frequency range.

**Figure 22:**
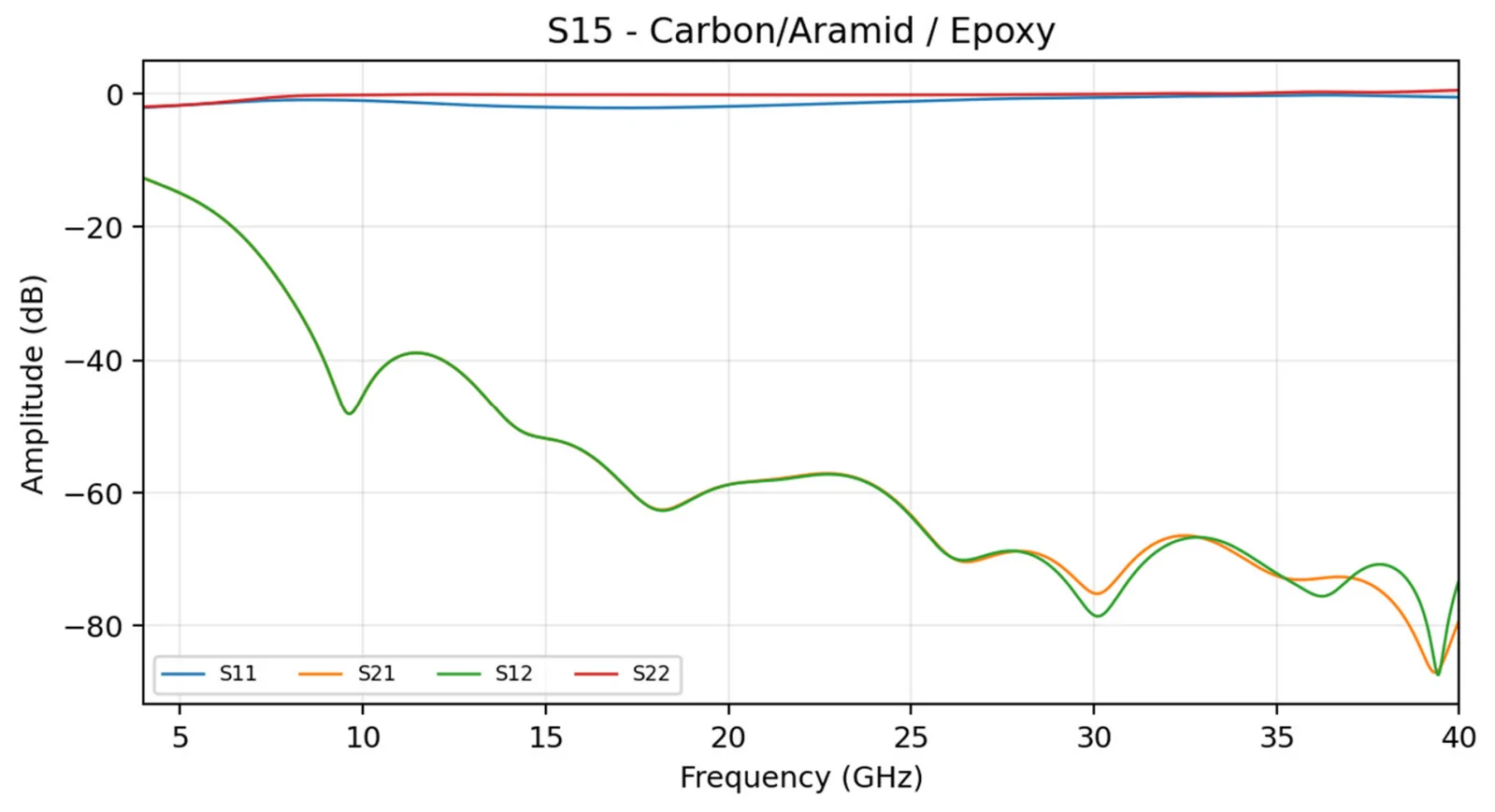
S_11_, S_21_, S_12_, and S_22_ curves of the S15 (carbon/aramid/epoxy) specimen over the 4–40 GHz frequency range.

**Figure 23:**
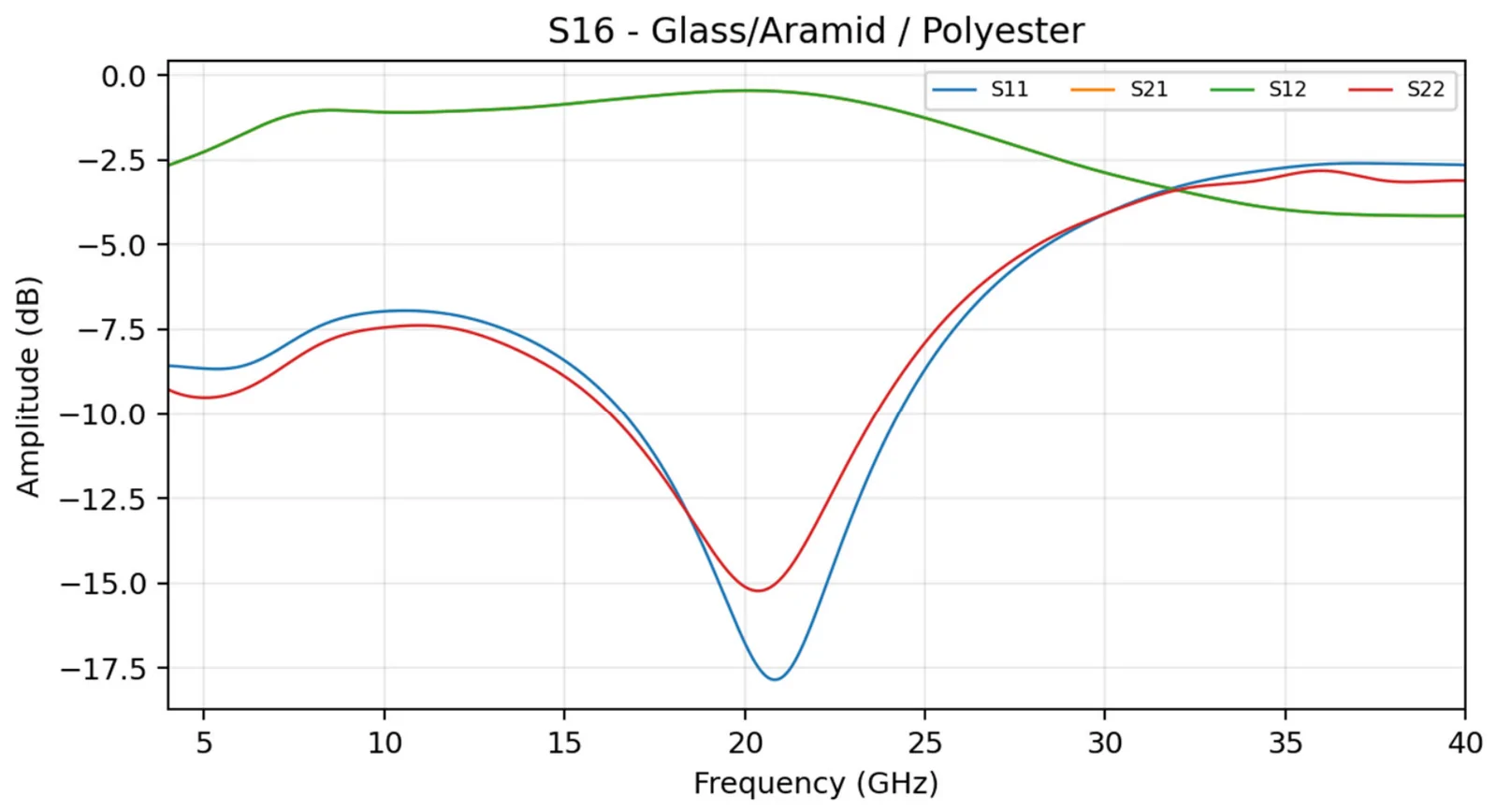
S_11_, S_21_, S_12_, and S_22_ curves of the S16 (glass/aramid/polyester) specimen over the 4–40 GHz frequency range.

**Figure 24:**
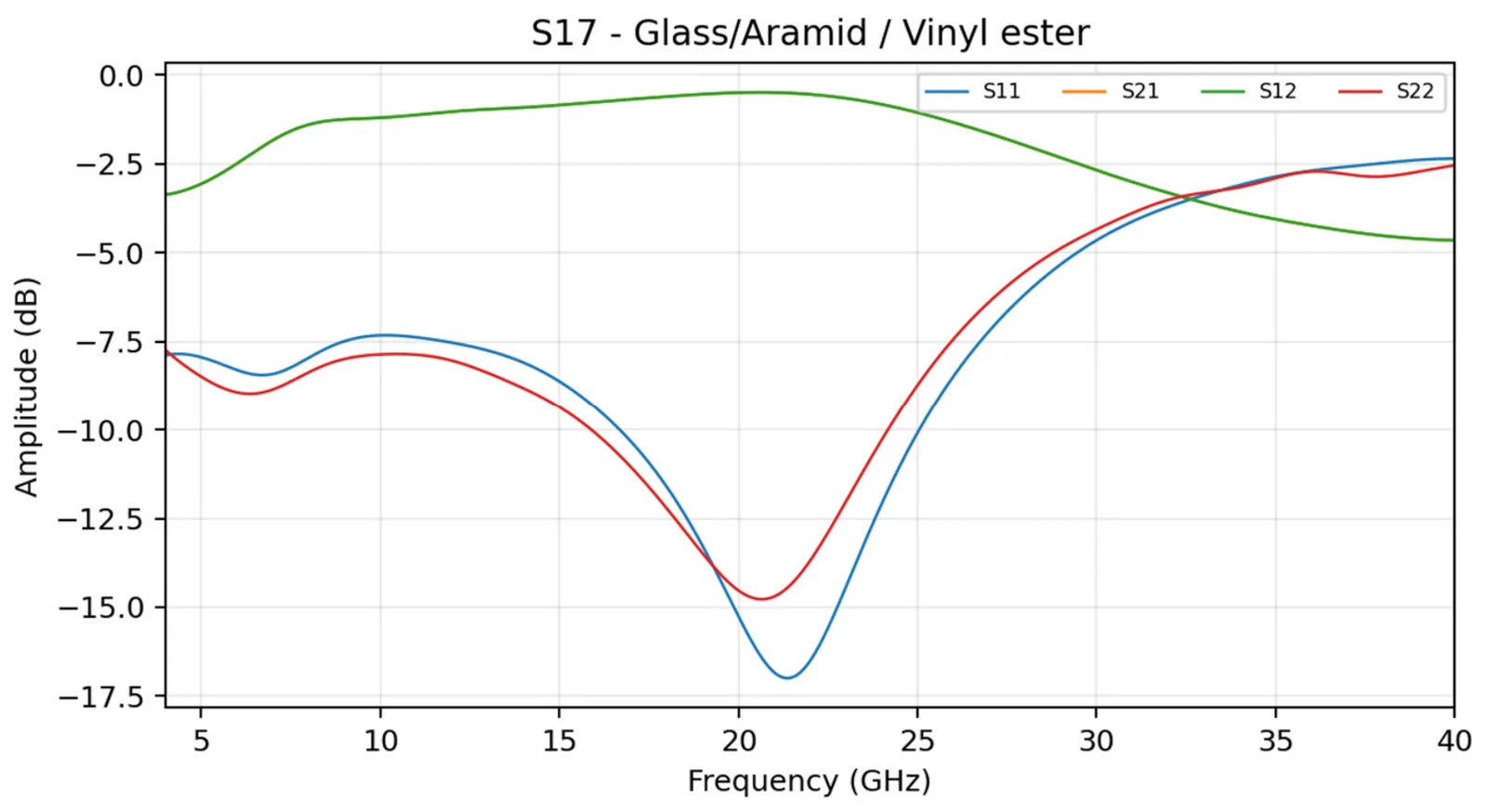
S_11_, S_21_, S_12_, and S_22_ curves of the S17 (glass/aramid/vinylester) specimen over the 4–40 GHz frequency range.

**Figure 25:**
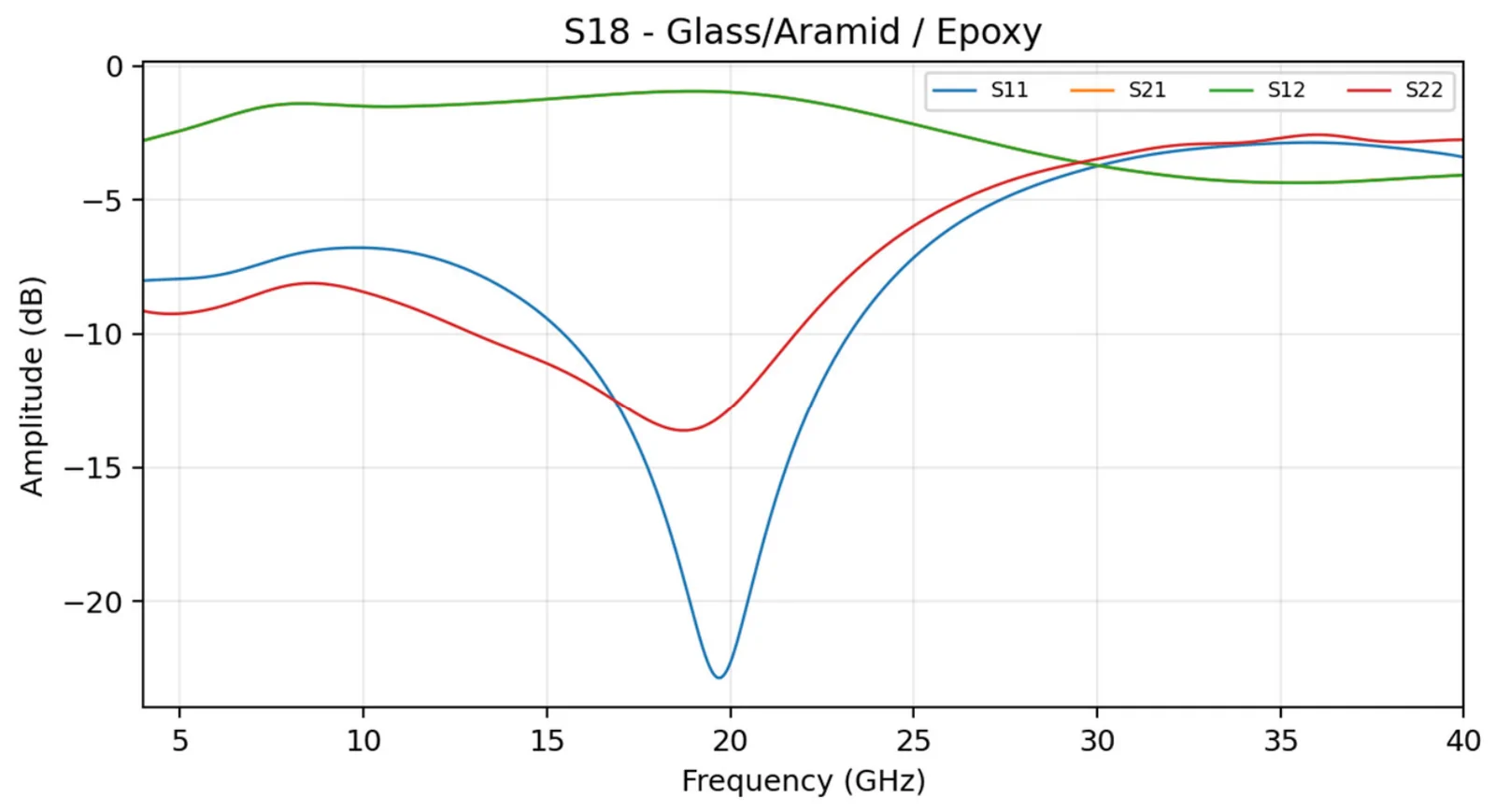
S_11_, S_21_, S_12_, and S_22_ curves of the S18 (glass/aramid/epoxy) specimen over the 4–40 GHz frequency range.

**Figure 26:**
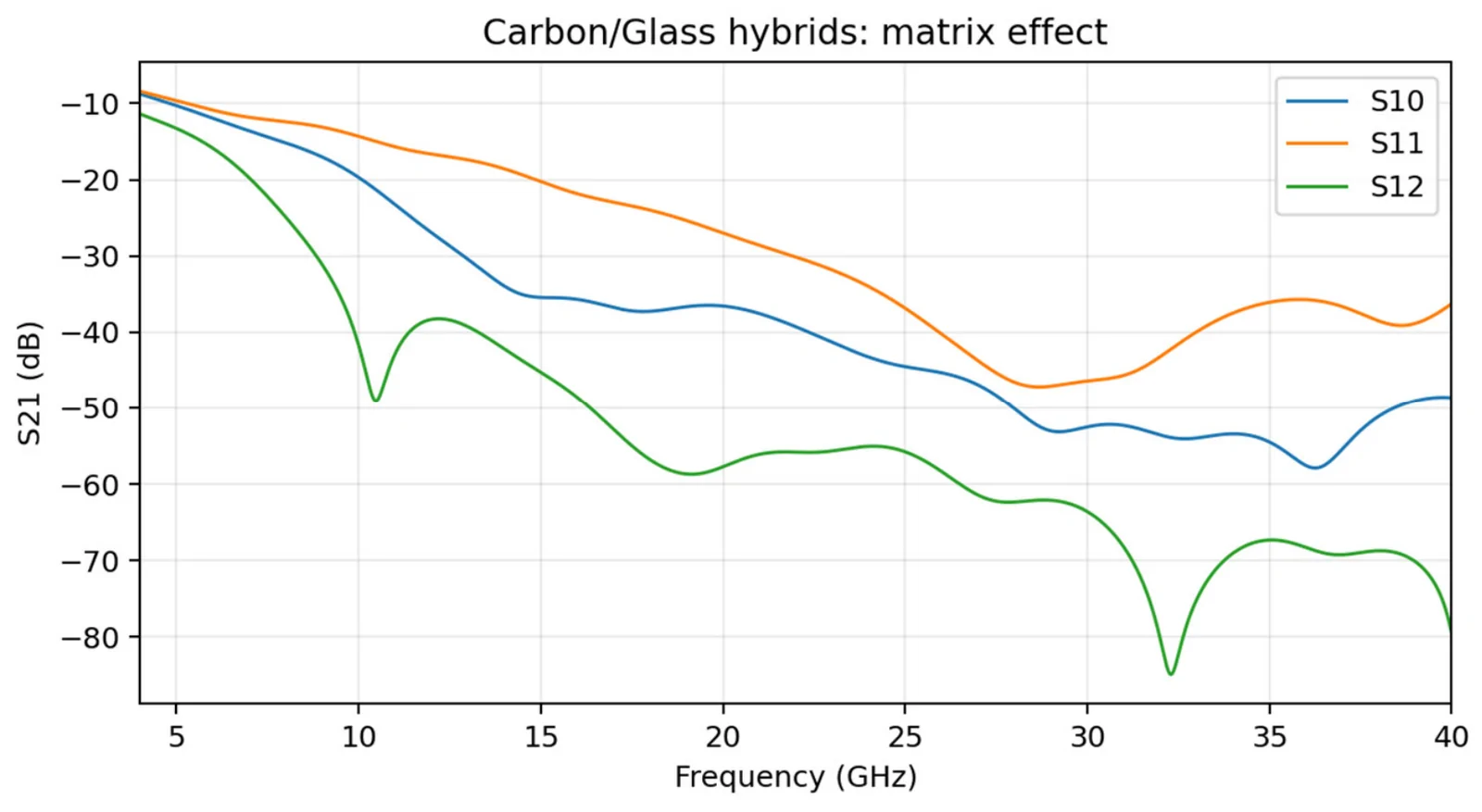
Comparison of the S_21_ curves for evaluating the matrix effect in carbon/glass hybrid composites.

**Figure 27:**
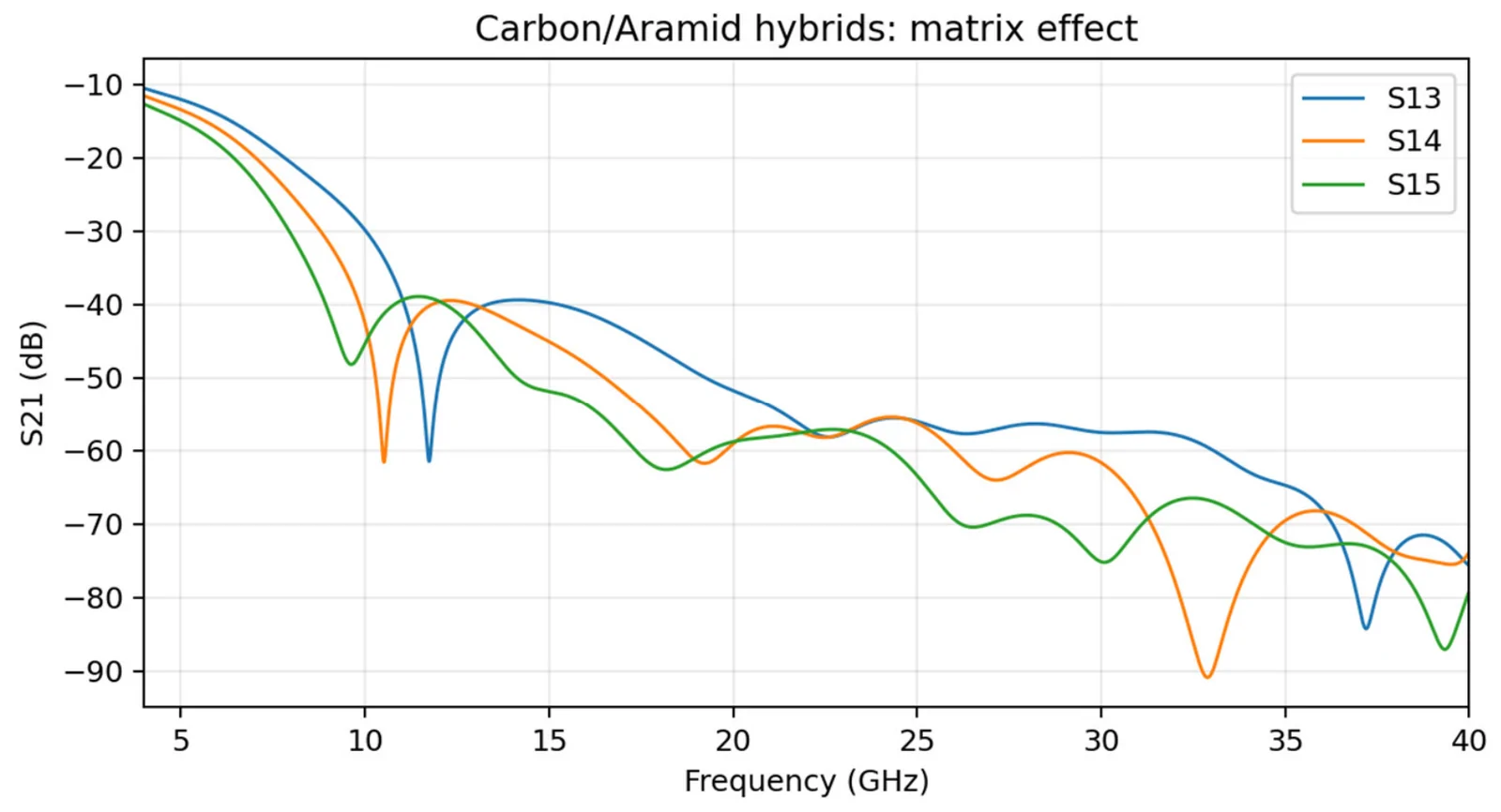
Comparison of the S_21_ curves for evaluating the matrix effect in carbon/aramid hybrid composites.

**Figure 28:**
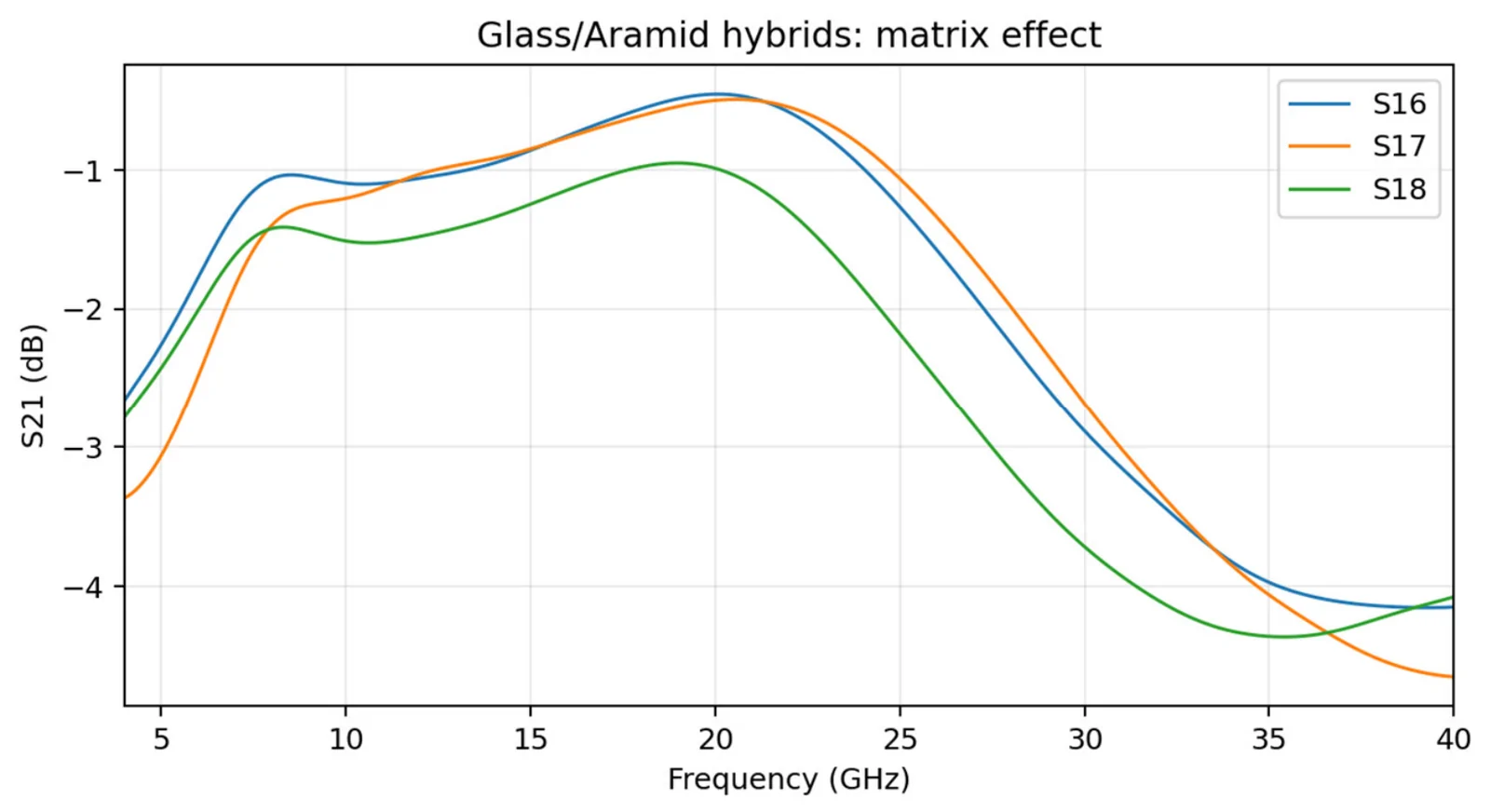
Comparison of the S_21_ curves for evaluating the matrix effect in glass/aramid hybrid composites.

**Figure 29:**
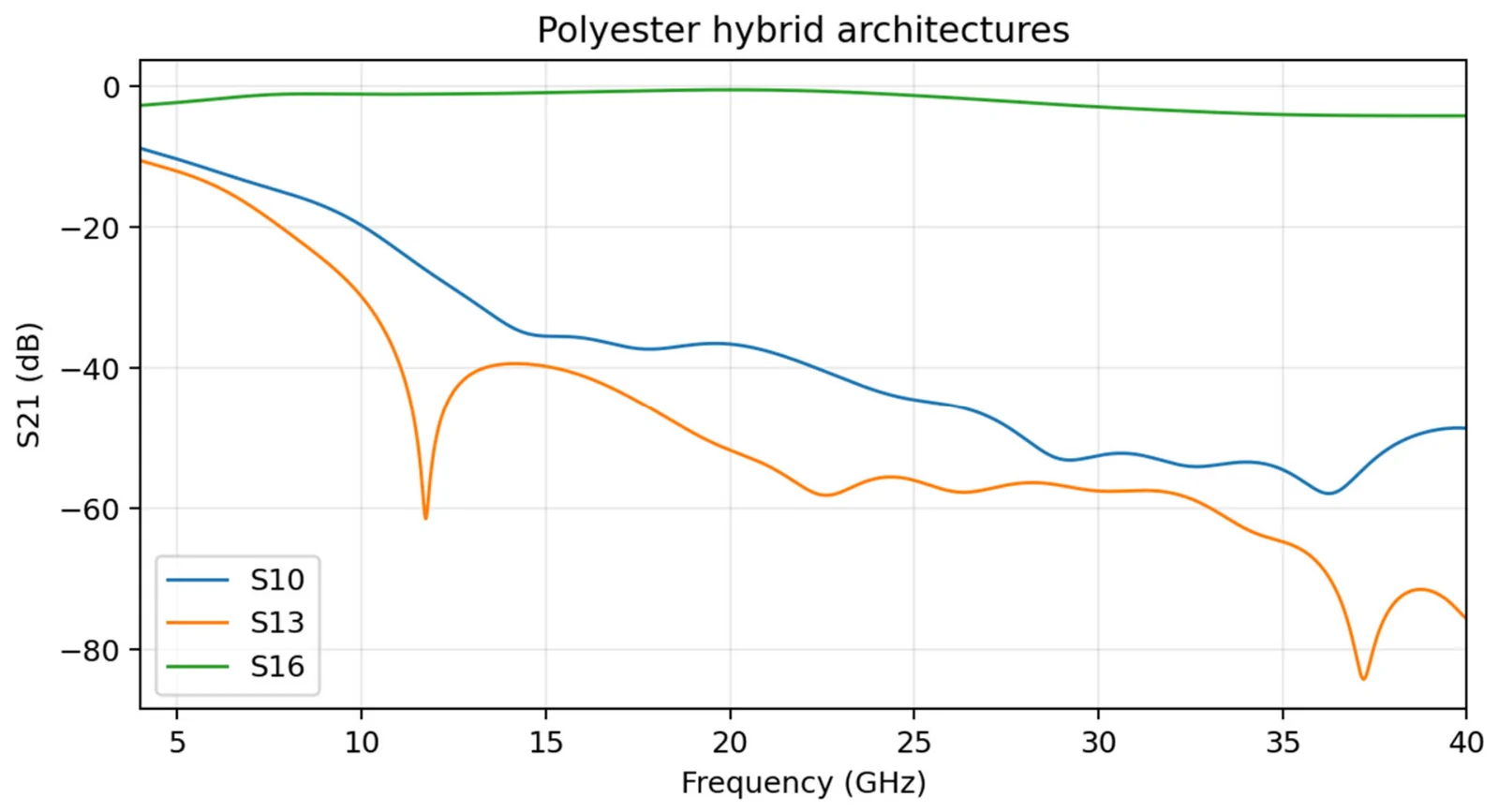
Comparison of the S_21_ curves for polyester matrix hybrid architectures.

**Figure 30:**
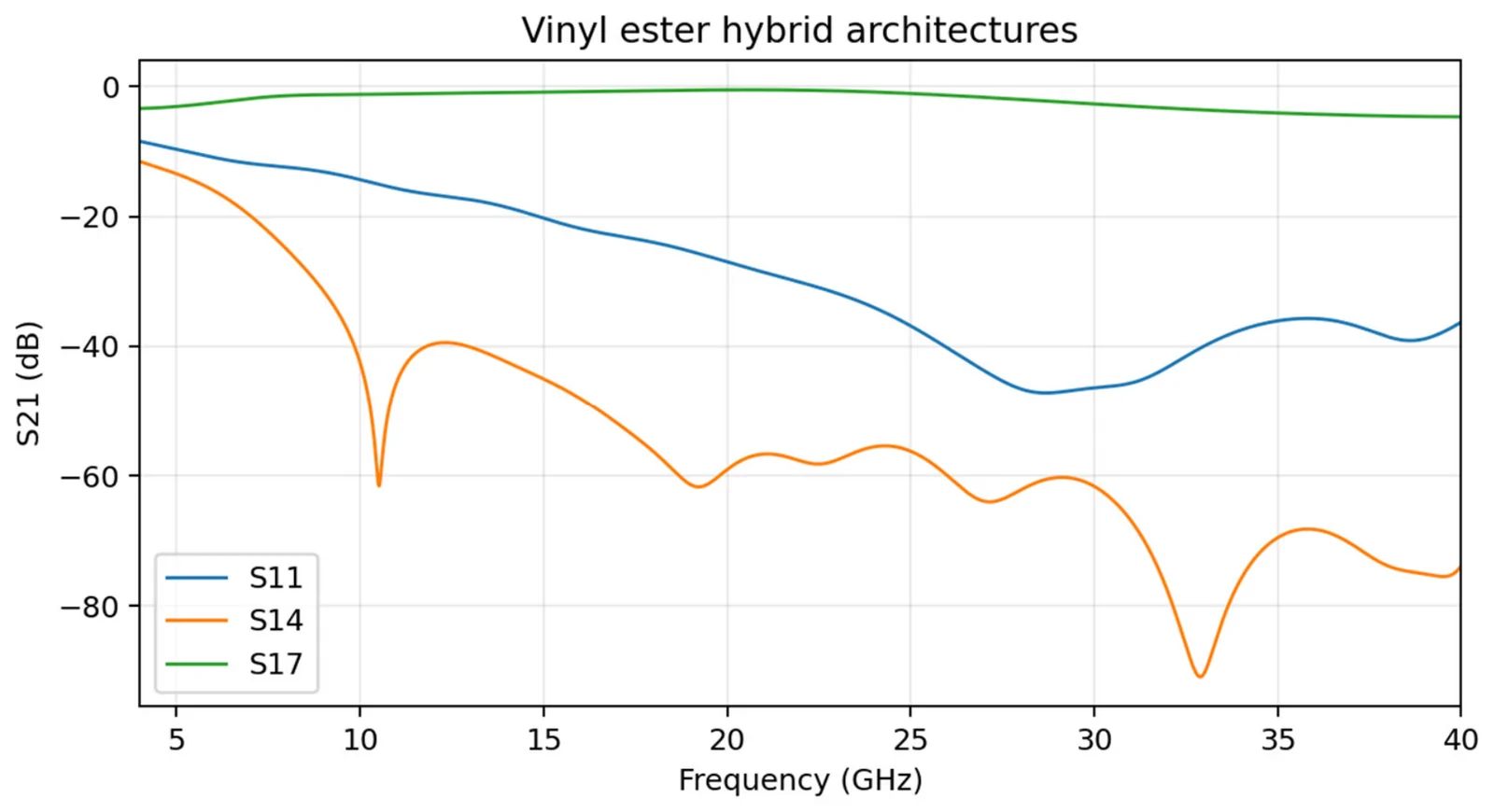
Comparison of the S_21_ curves for vinylester matrix hybrid architectures.

**Figure 31:**
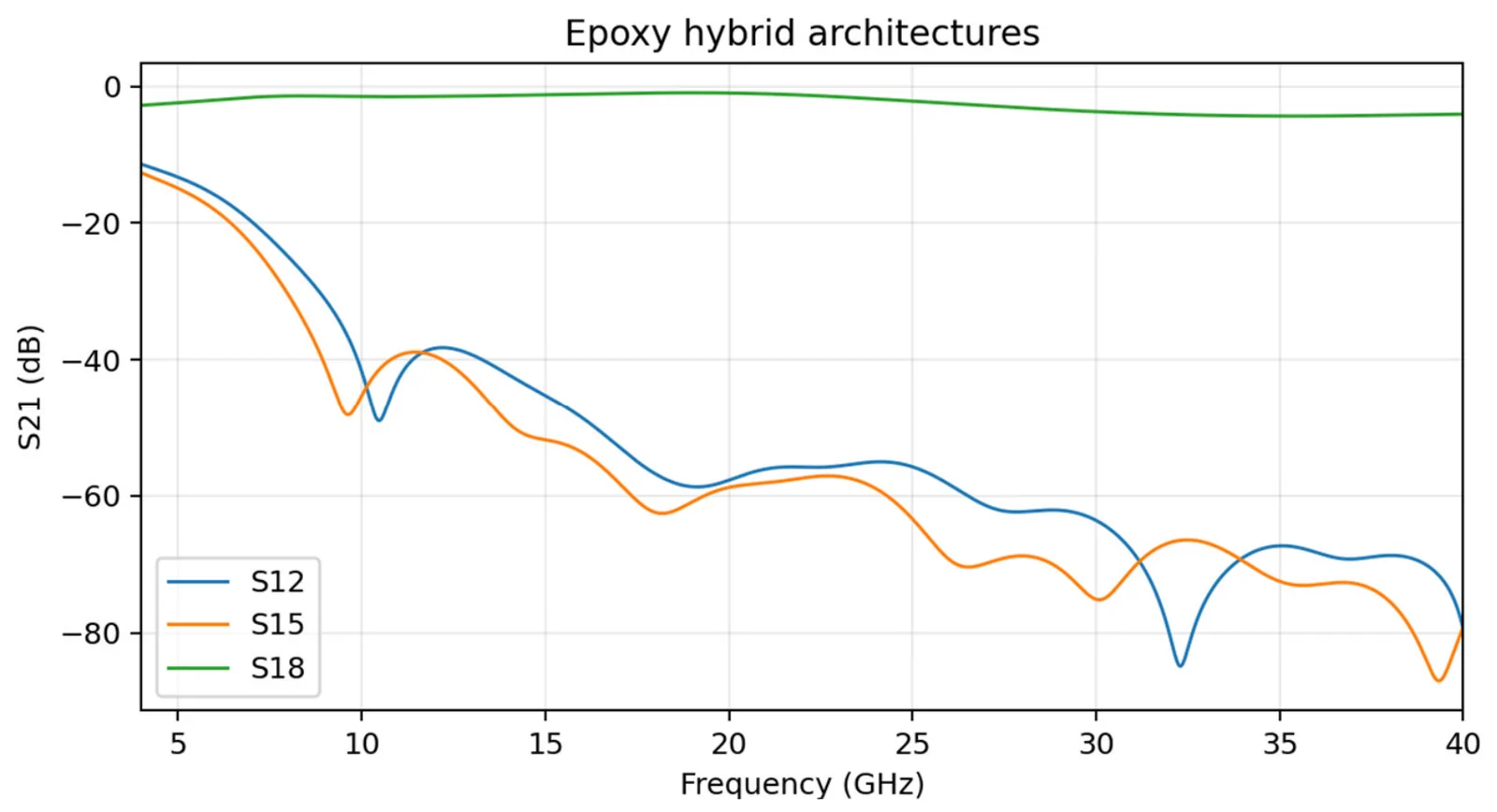
Comparison of the S_21_ curves for epoxy matrix hybrid architectures.

**Table 1:** Material Constituents and Configurations of the Composite Specimens.

Specimens					
Sample Numbers	Fiber	Core	Fiber	Resin	Thickness (mm)
S1	Carbon Fiber (2x)	Coremat	Carbon Fiber (2x)	Polyester	3.12 ± 0.08
S2	Carbon Fiber (2x)	Coremat	Carbon Fiber (2x)	Vinylester	3.10 ± 0.07
S3	Carbon Fiber (2x)	Coremat	Carbon Fiber (2x)	Epoxy	3.08 ± 0.05
S4	Glass fiber (2x)	Coremat	Glass fiber (2x)	Polyester	3.45 ± 0.10
S5	Glass fiber (2x)	Coremat	Glass fiber (2x)	Vinylester	3.40 ± 0.06
S6	Glass fiber (2x)	Coremat	Glass fiber (2x)	Epoxy	3.38 ± 0.07
S7	Aramid fiber (2x)	Coremat	Aramid fiber (2x)	Polyester	3.25 ± 0.09
S8	Aramid fiber (2x)	Coremat	Aramid fiber (2x)	Vinylester	3.22 ± 0.08
S9	Aramid fiber (2x)	Coremat	Aramid fiber (2x)	Epoxy	3.18 ± 0.06
S10	Carbon Fiber (2x)	Coremat	Glass fiber (2x)	Polyester	3.30 ± 0.09
S11	Carbon Fiber (2x)	Coremat	Glass fiber (2x)	Vinylester	3.26 ± 0.07
S12	Carbon Fiber (2x)	Coremat	Glass fiber (2x)	Epoxy	3.24 ± 0.06
S13	Carbon Fiber (2x)	Coremat	Aramid fiber (2x)	Polyester	3.18 ± 0.08
S14	Carbon Fiber (2x)	Coremat	Aramid fiber (2x)	Vinylester	3.15 ± 0.07
S15	Carbon Fiber (2x)	Coremat	Aramid fiber (2x)	Epoxy	3.12 ± 0.05
S16	Glass fiber (2x)	Coremat	Aramid fiber (2x)	Polyester	3.35 ± 0.10
S17	Glass fiber (2x)	Coremat	Aramid fiber (2x)	Vinylester	3.31 ± 0.08
S18	Glass fiber (2x)	Coremat	Aramid fiber (2x)	Epoxy	3.28 ± 0.06

**Table 2:** Physical and compositional properties of the composite specimens.

Sample Code	Matrix Resin Type	Fiber Reinforcement Architecture (Outer/Inner Layers)	Average Thickness, t ± SD (mm)	Experimental Density, ρexp (g/cm^3^)	Fiber Mass Fraction, Wf (%)	Resin Mass Fraction, Wm (%)	Fiber Volume Fraction, Vf (%)	Estimated Void Fraction, Vv (%)
S1	Polyester	Carbon/Carbon	3.12 ± 0.08	1.18	28.5	71.5	21.2	3.2
S2	Vinylester	Carbon/Carbon	3.10 ± 0.07	1.20	29.1	70.9	21.8	3.1
S3	Epoxy	Carbon/Carbon	3.08 ± 0.05	1.21	29.8	70.2	22.5	2.5
S4	Polyester	Glass/Glass	3.45 ± 0.10	1.38	41.2	58.8	24.8	3.8
S5	Vinylester	Glass/Glass	3.40 ± 0.06	1.40	42.5	57.5	25.6	3.0
S6	Epoxy	Glass/Glass	3.38 ± 0.07	1.42	43.1	56.9	26.1	2.8
S7	Polyester	Aramid/Aramid	3.25 ± 0.09	1.15	31.0	69.0	24.2	3.6
S8	Vinylester	Aramid/Aramid	3.22 ± 0.08	1.17	31.8	68.2	24.9	3.2
S9	Epoxy	Aramid/Aramid	3.18 ± 0.06	1.19	32.5	67.5	25.5	2.7
S10	Polyester	Carbon + Glass Hybrid	3.30 ± 0.09	1.28	35.2	64.8	23.1	3.5
S11	Vinylester	Carbon + Glass Hybrid	3.26 ± 0.07	1.30	36.0	64.0	23.8	3.0
S12	Epoxy	Carbon + Glass Hybrid	3.24 ± 0.06	1.31	36.8	63.2	24.4	2.6
S13	Polyester	Carbon + Aramid Hybrid	3.18 ± 0.08	1.16	29.8	70.2	22.8	3.4
S14	Vinylester	Carbon + Aramid Hybrid	3.15 ± 0.07	1.18	30.5	69.5	23.4	3.1
S15	Epoxy	Carbon + Aramid Hybrid	3.12 ± 0.05	1.20	31.2	68.8	24.0	2.6
S16	Polyester	Glass + Aramid Hybrid	3.35 ± 0.10	1.26	36.5	63.5	24.5	3.7
S17	Vinylester	Glass + Aramid Hybrid	3.31 ± 0.08	1.28	37.2	62.8	25.1	3.2
S18	Epoxy	Glass + Aramid Hybrid	3.28 ± 0.06	1.29	38.0	62.0	25.8	2.8

**Table 3:** Maximum tensile strength and Tukey HSD results for the composite configurations.

Sample	Maximum Tensile Strength (MPa), Mean ± SD	Significantly Different from (Tukey HSD, *p* < 0.05)
S1	169.4 ± 11.6	S3, S7, S14, S15
S2	182.5 ± 12.2	S3, S7, S14, S15, S16, S18
S3	231.2 ± 16.6	S1, S2, S4–S14, S16–S18
S4	169.01 ± 11.6	S3, S7, S14, S15
S5	182.1 ± 11.5	S3, S7, S14, S15, S16, S18
S6	156.1 ± 10.5	S3, S7, S15
S7	106.3 ± 7.1	S1–S6, S8–S13, S15, S17–S18
S8	150.3 ± 10.3	S3, S7, S15
S9	179.7 ± 12.0	S3, S7, S14, S15
S10	153.1 ± 10.3	S3, S7, S15
S11	145.9 ± 9.9	S3, S7, S15
S12	171.9 ± 11.4	S3, S7, S14, S15
S13	144.10 ± 9.7	S3, S7, S15
S14	114.5 ± 7.2	S1, S2, S3, S4, S5, S9, S12, S15
S15	212.9 ± 15.8	S1, S2, S4–S14, S16–S18
S16	138.9 ± 9.3	S2, S3, S5, S7, S15
S17	147.1 ± 9.9	S3, S7, S15
S18	143.7 ± 9.6	S2, S3, S5, S7, S15

**Table 4:** One-way ANOVA results for maximum tensile strength.

Source of Variation	df	Sum of Squares (SS)	Mean Square (MS)	F	*p*-Value	η^2^	ω^2^
Between configurations	17	92,815.63	5459.74	35.340	<0.001	0.893	0.866
Within configurations (Error)	72	11,123.54	154.49	—	—	—	—
Total	89	103,939.17	—	—	—	—	—

Levene’s test: *p* = 0.969; α = 0.05. Table note: df, degrees of freedom; SS, sum of squares; MS, mean square; F, ANOVA F-statistic; *p*, probability value; η^2^, eta-squared; ω^2^, omega-squared. Statistical significance was accepted at *p* < 0.05.

**Table 5:** Mechanical Properties of the Fabricated Composite Structures Obtained from Three-Point Bending Tests and Tukey HSD post hoc results.

Sample	Maximum Force (N)	Maximum Displacement (mm)	Maximum Flexural Strain (%)	Maximum Flexural Stress (MPa), Mean ± SD	Significantly Different from (Tukey HSD, *p* < 0.05)
S1	178.8	1.65	1.16	134.0 ± 7.1	S2, S5, S6–S11, S13–S18
S2	179.3	1.13	0.88	147.1 ± 4.2	S1, S5–S18
S3	210.6	1.75	1.09	136.6 ± 6.8	S5–S11, S13–S18
S4	199.4	2.41	1.54	136.2 ± 6.8	S5–S11, S13–S18
S5	236.8	2.49	1.61	160.4 ± 8.0	S1–S4, S6–S18
S6	214.3	3.02	1.62	117.4 ± 5.9	S1–S5, S7–S9, S16–S18
S7	108.7	7.87	5.62	81.8 ± 4.1	S1–S15, S18
S8	68.7	1.17	0.92	55.5 ± 2.7	S1–S18, S8
S9	145.6	2.80	1.90	102.7 ± 5.1	S1–S9, S12, S13, S15–S17
S10	166.8	2.02	1.30	113.7 ± 5.7	S1–S5, S7, S8, S16–S18
S11	159.3	2.19	1.44	109.1 ± 5.4	S1–S5, S7, S8, S12, S16–S18
S12	203.1	2.21	1.34	125.4 ± 6.3	S2, S5, S7–S9, S11, S16–S18
S13	164.3	1.82	1.29	120.3 ± 6.0	S1–S5 S2, S7–S9, S16–S18
S14	156.2	1.88	1.34	113.1 ± 5.6	S1–S5, S7, S8, S16–S18
S15	172.5	4.19	2.76	116.30 ± 5.84	S1–S5, S7–S9, S16–S18
S16	118.7	5.14	3.25	78.80 ± 3.96	S1–S6, S8–S15, S18
S17	108.1	2.14	1.40	74.20 ± 3.73	S1–S6, S8–S15, S18
S18	154.3	3.59	2.18	94.80 ± 4.76	S1–S8, S10–S17

**Table 6:** Two-way ANOVA results for maximum flexural stress.

Source of Variation	df	Sum of Squares (SS)	F	*p*-Value	Partial ηp^2^
Matrix Resin Type	2	549.36	8.65	<0.001	0.194
Fiber Reinforcement Architecture	5	50,153.27	315.74	<0.001	0.956
Matrix × Architecture	10	12,177.42	38.33	<0.001	0.842
Residual	72	2287.37	—	—	—

Table note: df = degrees of freedom; SS = sum of squares; F = ANOVA F-statistic; *p* = probability value; ηp^2^ = partial eta-squared (effect size). Statistical significance was defined as *p* < 0.05. Five measurements were analyzed for each configuration (n = 5; N = 90).

**Table 7:** Heat Deflection Temperature (HDT) and Deflection Values of the Composite Specimens.

Sample	HDT (°C)	Deflection (mm)	Sample	HDT (°C)	Deflection (mm)
S1	75.2	0.55	S10	71.7	0.45
S2	78.6	0.55	S11	73	0.45
S3	61.9	0.55	S12	57.8	0.41
S4	78.5	0.43	S13	76.2	0.55
S5	128	0.45	S14	80.7	0.55
S6	59.2	0.38	S15	72.3	0.49
S7	73.7	0.55	S16	73.5	0.49
S8	71.8	0.72	S17	71.4	0.65
S9	70.1	0.60	S18	66.9	0.76

**Table 8:** Vicat Softening Temperature Test Results of the Hybrid Composite Specimens.

Sample	VST (°C)	Sample	VST (°C)
S1	82.1	S10	74.3
S2	120.4	S11	87.9
S3	71.5	S12	86.2
S4	112.2	S13	108.4
S5	132.7	S14	125.9
S6	58.8	S15	131.5
S7	91.3	S16	69.9
S8	74.1	S17	67.9
S9	74.4	S18	72

**Table 9:** Calculated Average Electromagnetic Power Coefficients and Shielding Parameters for Specimens S1–S18.

Specimen	Reinforcement	Matrix	R_1_ (%)	T_21_ (%)	A_1_ (%)	SE_T_ (dB)	A_eff_ (%)
S1	Carbon	Polyester	95.11	0.26	4.62	25.72	94.52
S2	Carbon	Vinylester	94.37	0.17	5.46	27.70	96.98
S3	Carbon	Epoxy	94.25	0.25	5.49	25.94	95.57
S4	Glass	Polyester	32.33	62.42	5.25	2.05	7.75
S5	Glass	Vinylester	29.23	63.56	7.20	1.97	10.17
S6	Glass	Epoxy	31.95	58.87	9.17	2.30	13.48
S7	Aramid	Polyester	14.98	73.38	11.63	1.34	13.68
S8	Aramid	Vinylester	13.90	73.19	12.90	1.36	14.98
S9	Aramid	Epoxy	18.52	69.02	12.45	1.61	15.29
S10	Carbon/Glass	Polyester	91.63	0.93	7.44	38.45	52.33
S11	Carbon/Glass	Vinylester	79.45	1.61	18.93	17.91	92.12
S12	Carbon/Glass	Epoxy	93.85	0.33	5.82	24.72	94.52
S13	Carbon/Aramid	Polyester	80.33	0.49	19.18	23.06	97.49
S14	Carbon/Aramid	Vinylester	80.56	0.32	19.11	24.84	98.31
S15	Carbon/Aramid	Epoxy	77.39	0.21	22.40	26.60	99.03
S16	Glass/Aramid	Polyester	24.95	66.85	8.20	1.75	10.92
S17	Glass/Aramid	Vinylester	23.57	65.96	10.47	1.81	13.70
S18	Glass/Aramid	Epoxy	25.18	59.44	15.38	2.26	20.56

**Table 10:** Standard deviation (SD) values of the measured S-parameters for the investigated composite configurations over the 4–40 GHz frequency range.

Sample	SD S_11_ (dB)	SD S_21_ (dB)	SD S_12_ (dB)	SD S_22_ (dB)	Mean SD (dB)
S1	0.05	0.14	0.14	0.05	0.09
S2	0.05	0.14	0.14	0.05	0.09
S3	0.05	0.14	0.14	0.05	0.09
S4	0.08	0.05	0.05	0.08	0.07
S5	0.08	0.05	0.05	0.08	0.07
S6	0.07	0.05	0.05	0.08	0.06
S7	0.07	0.05	0.05	0.07	0.06
S8	0.07	0.05	0.05	0.08	0.06
S9	0.07	0.05	0.05	0.08	0.06
S10	0.05	0.12	0.05	0.05	0.07
S11	0.05	0.11	0.11	0.05	0.08
S12	0.05	0.14	0.14	0.05	0.09
S13	0.05	0.14	0.14	0.05	0.09
S14	0.05	0.15	0.14	0.05	0.09
S15	0.05	0.14	0.14	0.05	0.09
S16	0.07	0.05	0.05	0.06	0.06
S17	0.07	0.05	0.05	0.06	0.06
S18	0.07	0.05	0.05	0.06	0.06

## Data Availability

The original contributions presented in this study are included in the article. Further inquiries can be directed to the corresponding author.
